# Multiple asters organize the yolk microtubule network during dclk2-GFP zebrafish epiboly

**DOI:** 10.1038/s41598-022-07747-7

**Published:** 2022-03-08

**Authors:** Maria Marsal, Matteo Bernardello, Emilio J. Gualda, Pablo Loza-Alvarez

**Affiliations:** ICFO-Institut de Ciencies Fotoniques, The Barcelona Institute of Science and Technology, Castelldefels, Barcelona, Spain

**Keywords:** Cell biology, Developmental biology, Imaging, Microscopy

## Abstract

It is known that the organization of microtubule (MT) networks in cells is orchestrated by subcellular structures named MT organizing centers (MTOCs). In this work, we use Light Sheet Fluorescence and Confocal Microscopy to investigate how the MT network surrounding the spherical yolk is arranged in the dclk2-GFP zebrafish transgenic line. We found that during epiboly the MT network is organized by multiple aster-like MTOCS. These structures form rings around the yolk sphere. Importantly, in wt embryos, aster-like MTOCs are only found upon pharmacological or genetic induction. Using our microscopy approach, we underscore the variability in the number of such asters in the transgenic line and report on the variety of global configurations of the yolk MT network. The asters’ morphology, dynamics, and their distribution in the yolk sphere are also analyzed. We propose that these features are tightly linked to epiboly timing and geometry. Key molecules are identified which support this asters role as MTOCs, where MT nucleation and growth take place. We conclude that the yolk MT network of dclk2-GFP transgenic embryos can be used as a model to organize microtubules in a spherical geometry by means of multiple MTOCs.

## Introduction

The organization of microtubules (MTs) is not random but governed by defined subcellular regions called MT organizing centers (MTOCs). Here, MTs are often arranged radially, are nucleated from a central organizing region, and form what is known as a MT aster. This is how the centrosomes, used by dividing cells, organize the mitotic spindle. Moreover, differentiated cells can reassign MTOC functions to non-centrosomal sites, and thus interphase MTs are also arranged in parallel arrays and in radial patterns. Additionally, MT-based spatial organization is influenced by cell size and cell geometry^1–3^.

The zebrafish (*Danio rerio*) embryo presents a spherical and large yolk cell of about 700–800 µm in diameter, similar in size to amphibian zygotes (~ 1200 µm), and far exceeding the size of standard somatic cells. Although initially anuclear, the yolk cell becomes a syncytium at around the 512-cell stage due to the collapse of the marginal blastomeres. These cells release their content into the yolk, forming what is known to be the yolk syncytial layer (YSL)^4^. This structure is adjacent to a thin yolk cytoplasmic layer (YCL), which wraps the viscous yolk mainly composed of lipid granules^5–7^. Following that, the process of epiboly occurs, consisting of the thinning and spreading of different cell layers over and around the spherical yolk cell^8,9^.

It is known that the zebrafish YSL and YCL contain MT networks that undergo various changes (MT density, MT length) over the different developmental stages^10–14^. Given its size and shape, the zebrafish yolk cell arises as an ideal model for studying how MT-based spatial organization scales with size, how it relates to geometrical constraints, and how its global architecture reshapes over time. With this aim, we have used Light-Sheet Fluorescence Microscopy (LSFM), confocal laser scanning microscopy (CLSM), and the zebrafish MT reporter line Tg(Xla.Eef1a1:dclk2a-GFP)^14^ (from now on noted as dclk2-GFP) to investigate the whole MT organization in the spherical yolk cell of these transgenic embryos during epiboly. In this work, we give a mesoscopic description of the spatio-temporal MT arrangement of the network and propose cellular and molecular mechanisms responsible for building this particular MT network.

Specifically, we identify the presence of undescribed MT asters in the YCL in the dclk2-GFP embryos, at middle-stage epiboly. We analyze their spatial dimensions, dynamics, and micron-scale architecture. We also show that MT asters appear within the YCL regularly in a precise time window, occupying the yolk surface, and assembling into large 3D patterns of radially oriented MTs. We quantify their position and observe that YCL asters do not randomly locate in the yolk sphere, but instead group at one or more spherical latitudes. We also address how the presence and number of YCL asters impact epiboly progression.

Remarkably, we describe that these yolk MT asters (YCL asters) are observed in the transgenic and over-expressing embryos [transient expression of the DCLK (doublecortin-like kinase) and its ortholog protein DCX (doublecortin)] but never form in wildtype embryos, unless they are incubated with Taxol, a MT stabilizer compound. Moreover, we find that different configurations, in terms of the variable number of YCL asters present in the transgenic embryos, are compatible with development. Therefore, the yolk MT network arises as an example of plasticity in development.

Those observations have been quantified across many embryos thanks to the increased throughput capabilities of our custom-made LSFM microscope^15^. Our approach makes it possible to look at the embryo as a whole, to visualize the dynamic organization in 3D with high resolution (0.65 µm) and contrast, and to quantify the phenotypic variability that has remained unseen up to now.

The dclk2-GFP zebrafish transgenic line has been used as MT reporter line in several publications^14–25^ to visualize MT dynamics in live cells. The Tg(ef1α:dclk-GFP) transgene contains two doublecortin domains (microtubule-binding domain) of zebrafish dclk1 fused to gfp, and under the control of the Xenopus ef1α promoter. In this work, we use it to image epiboly stages with LSFM, following a previous study in our lab by which the in toto observation of this specific transgenic line has allowed to underscore the dynamics of the MT network prior to epiboly stages^10^.

During epiboly, the differences in yolk MT organization observed across wildtype and within the transgenic dclk2-GFP embryos population allows us to recommend to carefully evaluate whether the conclusions arisen from the study of transgenic lines in general can be extrapolated to the wt population. In our case, though “unnatural”, this model presents advanced knowledge on the collective behaviour of MT in a sphere. Moreover, this is one of the few in vivo examples where the formation, growth and interaction of MT asters can be addressed, complementing previous in vitro studies^26,27^. We show that they are sites of MT nucleation and polymerization. Interestingly, the formation of these structures in a transgenic line reflects how labile MT networks can be and indicates that the zebrafish yolk MT network can be used as a 3D biological model of MT organization where MT arrays can reorient in response to different cues. Finally, the description of the structure's distribution in the spherical yolk surface sets the basis to study how MT organization is influenced by a spherical geometry.

## Results

### The yolk MT network in dclk2-GFP embryos displays multiple MT asters

**Figure 1:**
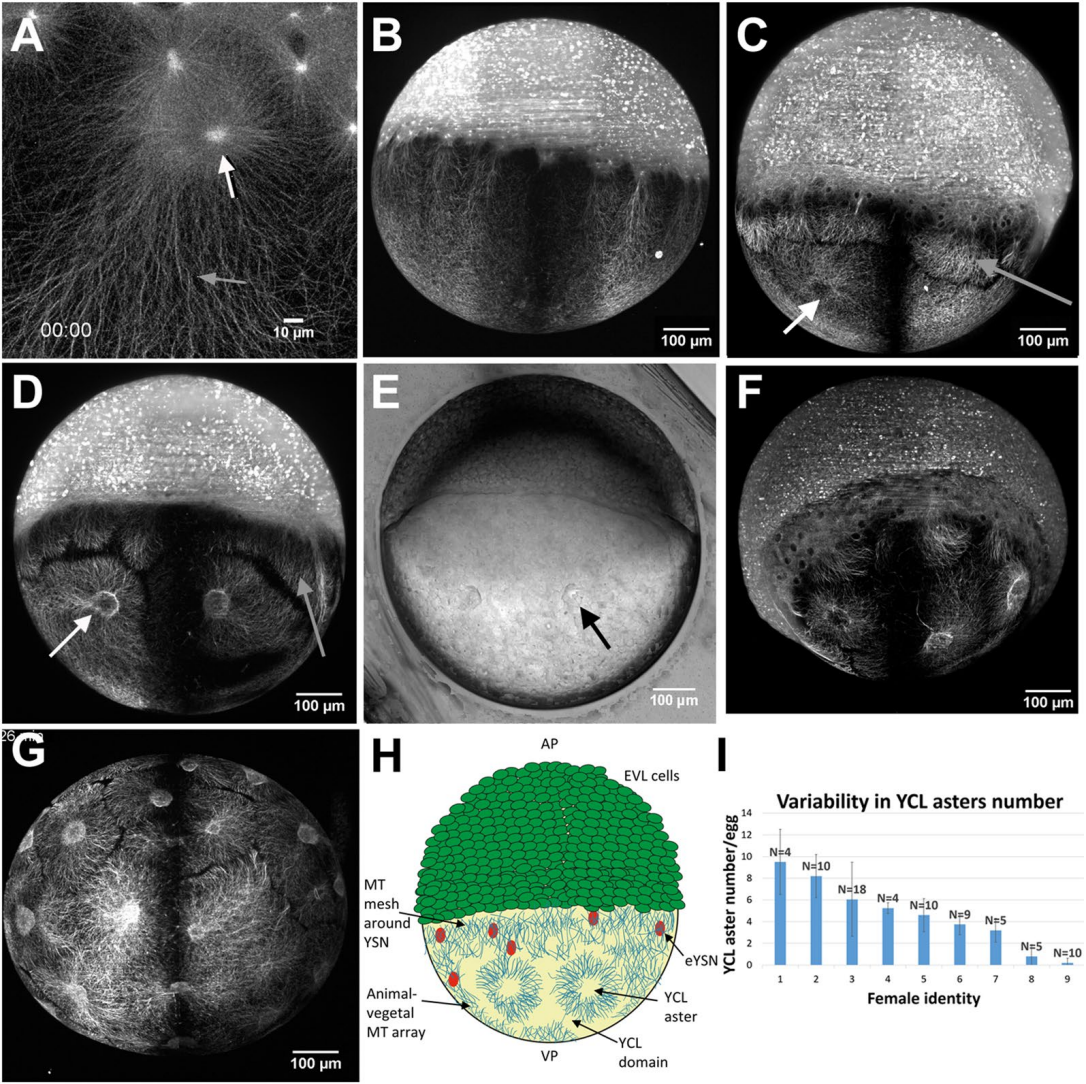
The variable presence of YCL asters in the yolk MT network of dclk2-GFP transgenic zebrafish embryos. (**A**) CLSM zoom into the YSL shows meshed interconnected MT with apparent spindles during mitosis of e-YSN (white arrow) and the AV parallel YCL MT arrays, emanating from MTOCs associated to the most vegetal e-YSN (grey arrow). (**B**–**F**) Lateral views of transgenic embryos, with the animal pole at the top and the vegetal pole at the bottom. (**B**) At sphere stage, parallel MT arrays emerge from the YSL, covering the YCL. At the animal pole, mitotic spindles of dividing cells are also visible. (**C**,**D**) Many embryos present YCL asters, a radial MT organization in defined regions (white arrow). These YCL asters organize MT in clear domains, different from the MT network emerging from the YSL (grey arrow), and they are also visible in (**E**) bright-field imaging (black arrow) and can be clearly observed in the 3D volume rendering in Video S1. (**F**) Example of an embryo showing 5 YCL asters. (**G**) Some embryos (here a vegetal view) show up to 22 YCL asters, covering the entire YCL. (**H**) Schematic for the new configuration found in the transgenic line, not in scale. AP stands for animal pole and VP stands for vegetal pole. YCL asters coexist with AV parallel MT arrays and YSN MT mesh around YSN. (**I**) Comparison between the average number of YCL asters in eggs of selected Tg dclk2-GFP females. N stands for the total number of eggs analysed for the different females. All images except (**A**) are LSFM images. (**H**) Created with Adobe Illustrator CS6 (http://www.adobe.com).

We have primarily used dclk2-GFP embryos as a MT reporter transgenic line to study yolk MT organization. Thanks to our custom-made high throughput multi-view light-sheet imaging approach^10,28^, we have access, with high temporal (0.3–1 volume/min) and spatial (0.65–1.3 µm) resolutions, to the whole embryonic sphere throughout zebrafish early developmental stages. Compared to CLSM, LSFM provides high signal-to-noise ratio (SNR) images more efficiently, with reduced photo-bleaching and photo-toxicity^29,30^. Additionally, our original high-throughput sample mounting solution based on fluidic sample loading^31^ permits us to study a large embryo population (over one hundred) in a highly reproducible way, preserving sample viability. This approach has allowed us to show the great phenotypic variability on the yolk MT network in these transgenic embryos. Some examples are displayed in Fig. 1.

For the present study, we have selected zebrafish at epiboly stages, in which the embryo is an almost perfect sphere. Through the dclk2-GFP line we can observe, before epiboly starts, the presence of the two types of yolk MTs organization already described in the 1990s^11,12^. Those are, firstly, meshed interconnected MTs covering the entire YSL corona, with apparent spindles during mitosis of YSN (Fig. 1A). Secondly, the animal-vegetal (AV) parallel arrays of MTs of the YCL, emanating from potential MTOCs associated with the most vegetal e-YSN and ending at different latitudes of the yolk cell (Fig. 1B)^12,13^. Additionally, we can also observe the recently reported third array of MT deep inside the yolk cell^10^.

At 50% epiboly, the blastoderm covers almost completely the YSN and their associated MT arrays. From this point, the possibility to inspect many embryos with our LSFM microscope revealed a large phenotypic variability in the MT organization within the yolk cell. Common to most of the analyzed dclk2-GFP embryos, the vegetal array of MTs organizes in clear and visible compartments that we call yolk cytoplasmic layer asters (YCL asters) (Fig. 1C–G), with MTs radially orienting from each of the domain's center. To the best of our knowledge, this is the first time that those structures are reported. Interestingly, a simple observation with bright-field microscopy also reveals invaginations of the yolk membrane at the sites of each YCL aster (Fig. 1E). A 3D render (Video S1) shows how those structures have a half-sphere shape. This newly found configuration can be schematized as displayed in Fig. 1H, together with the AV parallel MT arrays and the mesh of MT surrounding e-YSN. It is worth mentioning that these structures appear regardless of the different sample preparation schemes (embryo with or without chorion) (see Supplementary Fig. S1) and regardless of the imaging approach used (LSFM or CLSM). A first qualitative glimpse revealed that the number of YCL asters changes from embryo to embryo. As an example, an embryo with 5 observable YCL asters (Fig. 1F) and an embryo with 22 YCL asters (as extreme case) (Fig. 1G) are shown.

To understand the origin of this variability we sought to investigate if there was a correlation between each female progenitor and the number of asters in their progeny. We quantified the number of YCL asters per embryo through the offspring of nine selected females (Fig. 1I). The analysis was carried out over a time period that allowed to cross the females several times. In order to increase the statistical relevance, we combined data obtained from LSFM, CLSM, and brightfield inspections, screening up to 75 embryos.

We have observed that a similar number of YCL asters per embryo was conserved across different layings of a given female. Thus, females laying eggs with many YCL asters continued to lay eggs with many asters over time. The same applied to females displaying eggs with a few or an intermediate number of asters. Notably, the presence of YCL asters in the dclk2-GFP transgenic line is transmitted across generations and therefore does not prevent development, i.e., embryos grow and become fertile adults. Interestingly, the variation in the YCL aster number is also heritable and transmitted to the next generation.

To our knowledge, this variation has remained unseen up to now, mainly because of lack of a proper microscopy methodology. Despite this variable penetrance is probably not-natural but induced when using the Tol2 transgenesis method^32^ we consider there is a need to report this issue, given the fact that this is the most used MT zebrafish fluorescent reporter line in the community.

### YCL asters form in stabilized yolk MT networks

**Figure 2:**
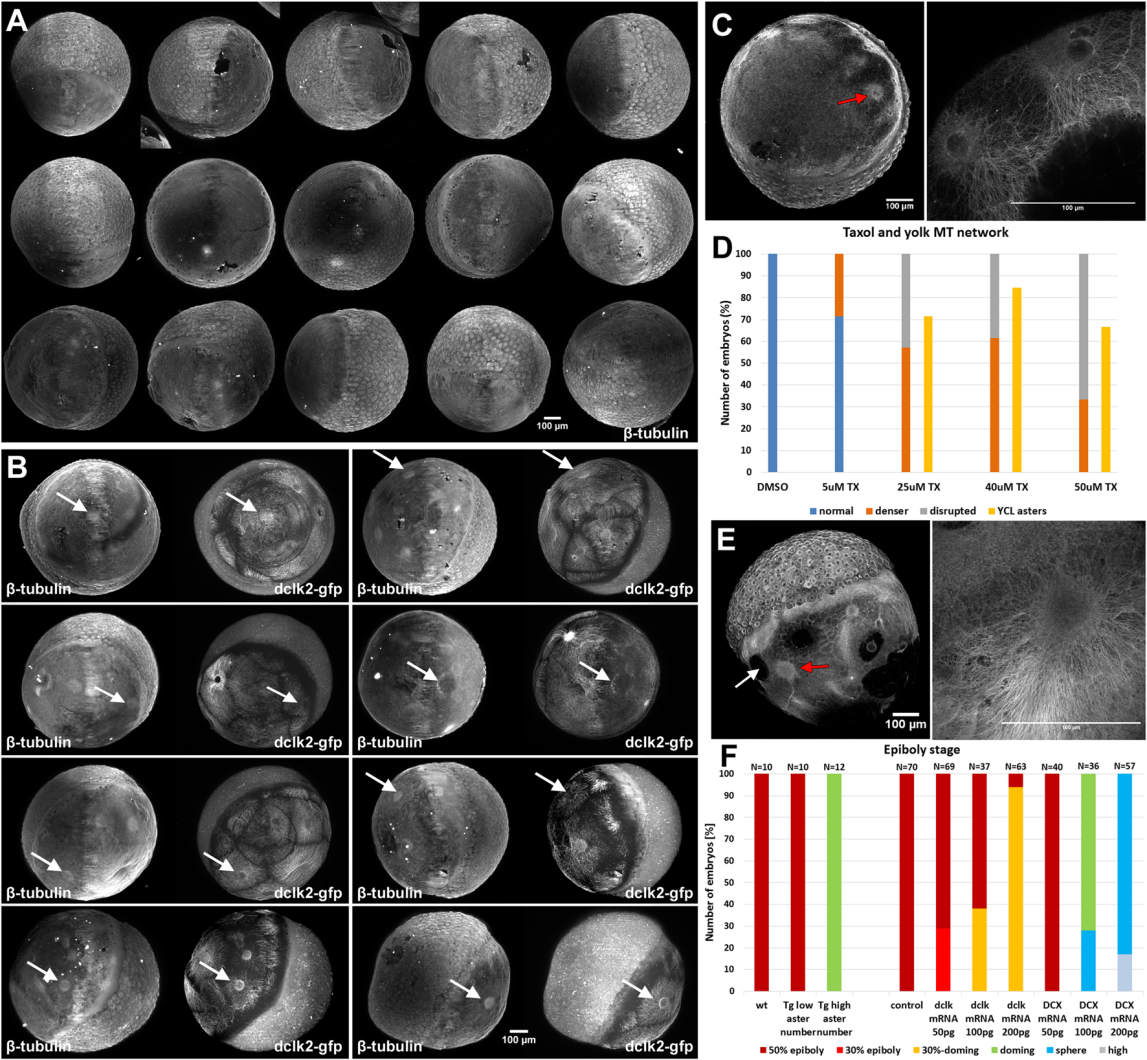
Natural and induced formation of YCL asters. (**A**,**B**) LSFM high-throughput screening of stained, previously fixed, embryos. (**A**) N = 15 wt embryos and (**B**) N = 8 dclk2-GFP embryos, immunostained against β-tubulin. On dclk2-GFP embryos, immunostaining reveals a high concentration of β-tubulin colocalizing with the dclk2-GFP signal in the YCL asters (white arrows in **B**). YCL asters are not visible in any wt embryo (**A**). (**C**) YCL asters form in the YCL upon dclk2-GFP overexpression in wt embryos. Ectopic asters appear in random positions (red arrow). Left: β-tubulin-stained embryo, low magnification vegetal view. Right: zoom in a couple of neighbouring asters. (**D**) Effect of Taxol on the organization of yolk MTs. Wt embryos were incubated with increasing drug concentrations from sphere stage. The yolk MT organization was evaluated in embryos fixed at 50% epiboly and immunostained for β-tubulin. The percentage of embryos with a denser (thick MTs) and a disrupted yolk MT network (MT bundles and MT free patches) increased progressively with higher drug concentration, compared to the normal organization of the yolk MT network in DMSO-incubated embryos (5 µM, N = 7; 25 µM, N = 7; 40 µM, N = 13; 50 µM, N = 12; DMSO, N = 15). Moreover, YCL asters appear from 25 µM Taxol dose. (TX: Taxol). (**E**) Representative wt embryo incubated with 40 µM Taxol and immunostained for β-tubulin. YCL asters appear in random positions (red arrow) and coexist with MT free regions (white arrow). Left: β-tubulin-stained embryo, low magnification lateral view. Right: zoom in one YCL aster. (**F**) Epiboly stage comparison between embryos of different conditions. Transgenic embryos with a high number of asters are delayed compared to their transgenic siblings having a small number of asters and compared to wt sibling embryos (first three columns in the graph). When control embryos (DMSO injected) are compared to *dclk2-gfp* and *DCX-gfp* mRNA injected embryos, epiboly delay correlates with the level of proteins overexpression (seven last columns in the graph).

The above-mentioned results indicate that in our model, the vast spherical yolk MT network is organized by means of multiple YCL asters, variable in number during epiboly stages. Next, we sought to understand whether the YCL asters feature was a particularity of the dclk2-GFP transgenic line. With this aim, we conducted a β-tubulin staining in both transgenic and wt embryos and performed a high-throughput screening with our custom-made LSFM (Fig. 2A,B). This immunostaining was expected to highlight MTs and MTOCs in the whole embryo. YCL asters were clearly highlighted with the β-tubulin staining in the dclk2-GFP transgenic embryos, but we were unable to find YCL asters in wt embryos. Thus, the yolk MT network organization mediated by multiple YCL asters appears a particular mechanism used by the dclk2-GFP transgenic line.

A set of experiments were designed to gain insight into the molecular mechanism responsible for this specific type of MT network organization and to understand the cause of the difference between the MT yolk network organization of dclk2-GFP and wt embryos. Suspecting a cause-effect relationship between amount of dclk2 protein and aster formation, we carried out a dosage-dependent assay and decided to overexpress either dclk or DCX proteins on wt embryos. Gain-of-function experiments have successfully been done before^33,34^. We performed mRNA injections at one-cell stage at different concentrations. The injected constructs were *dclk2-gfp* and the corresponding construct for the paralog gene doublecortin (DCX), *DCX-gfp*, that code for a GFP fusion to these two MT associated proteins, important for MT stabilization and nucleation^35,36^. Interestingly, the injection of 100 pg *dclk2-gfp* mRNA dose is able to induce the formation of YCL asters (Fig. 2C and Supplementary Fig. S2C), as also revealed with an immunostaining against β-tubulin. Injection of 200 pg dose induces bigger YCL asters. Asters can also be produced already in the presence of low levels of *DCX-gfp* mRNA (50 pg) (Supplementary Fig. S2D). YCL asters appear in random positions on the yolk surface.

The above experiments demonstrate a correlation between genetic overexpression and YCL asters formation and suggest that different transgenes' levels could cause the variability in YCL asters number observed in the transgenic line (Fig. 1I). Overexpressing dclk2 or DCX proteins by means of mRNA injection into the embryos is sufficient to induce YCL asters formation in wt embryos.

To further understand the molecular mechanism involved in the formation of YCL asters, we decided to manipulate MT dynamics in wt embryos by a different approach. With this aim, we used Taxol, a MT stabilizing drug, known to induce the formation of multiple MT asters in various scenarios^37–42^, commonly cell-free systems. Our intention was to generate asters in an in vivo system, the zebrafish yolk cell, in an effort to mimic YCL asters of dclk2-GFP transgenic embryos. Taxol promotes the MT polymerization process and prevents MT depolymerization by reducing the critical concentration of tubulin dimers and by enhancing the probability of spontaneous MT nucleation^37^. Wt embryos were incubated from sphere stage in different drug concentrations (sibling embryos were randomly divided into the different groups) and evaluated through immunostaining against β-tubulin. As previously shown^11–13^, Taxol treatment affects yolk cell MT organization and delays epiboly movements in zebrafish. Our experiments confirmed these results and showed that increasing Taxol concentrations amplifies the percentage of embryos exhibiting a yolk MT network with thick bundles [*denser* (orange in Fig. 2D)] and with gaps [*disrupted* (grey in Fig. 2D)]. Gaps are highlighted with a white arrow in Fig. 2E and MT bundles measured on average 8.60 ± 4.029 µm (N = 17). Moreover, we successfully induced the formation of obvious YCL asters (yellow in Fig. 2D) from 25 μM Taxol concentration, as displayed in Fig. 2E.

In conclusion, increasing the levels of these two closely related members of the MT nucleating proteins family or using Taxol to stabilize MTs, modifies the configuration of the wt yolk MT network. Under these conditions, the wt yolk MT network, normally composed of MTs uniformly covering the yolk cell, re-organizes into a multi-aster MT network.

The combination of the above-mentioned results allows to scale down the options to explain the mechanism that leads to the different organization of the yolk MT in wt vs dclk2-GFP transgenic embryos. We suggest that YCL asters are present in the transgenic line due to an excess of dclk2 protein. This excess might have a consequent overall increase of MT stabilization and/or generation of multiple nucleation sites in the yolk. This is supported by the fact that, in our hands, another protein (DCX) with known MT nucleating and stabilizing roles (Fig. 2C and Supplementary Fig. S2) and Taxol incubation (Fig. 2E) have a very similar effect. Future experiments may help to understand if the two functions (MT nucleation and stabilization) are required, if only one suffices, or if eventually one triggers the other.

To further investigate MT dynamics within YCL asters we performed a functional analysis with nocodazole, a chemical that interferes with microtubule polymerization. In an effort to confine the effect of the drug to the yolk, we injected it at the YSL at 1000 cell-stage, when the blastoderm has lost cytoplasmic bridges with the yolk cell. The yolk cell MT network of treated embryos exhibits areas devoid of MTs that coexist with YCL asters (see Supplementary Fig. S3 and Video S2). A first observation indicates that epiboly of the YSN is halted and that the movement of blastoderm is slowed down as reported by Solnica and Driever 1994 (Video S2). Interestingly, YCL asters resisted nocodazole action and persisted in the treated embryos up to the time the yolk cell bursted and the embryo degenerated. Instead, the response of e-YSN centrosome-nucleated microtubules was very different and this MT array was completely disrupted in the nocodazole treated embryos (see Supplementary Fig. S3). We hypothesize that Dclk2-GFP YCL asters contain a population of stable MT that are unsensitive to nocodazole effect, as it happens in taxol-induced asters in frog egg extracts^40^, cultured cells^37^ or astral MT in living sea urchin zygotes^43^, composed of at least a fraction of stable MTs.

### Asters impact on epiboly progression

Despite dclk2-GFP embryos develop and become fertile adults, we wanted to understand if the presence of YCL asters and its variable number had an influence on epiboly progression. With this aim, we monitored epiboly extension of the progeny of different transgenic fish and wt embryos. We found that epiboly is slower in dclk2-GFP eggs with more YCL asters and that this delay persists throughout epiboly (Fig. 2F and Supplementary Fig. S2A,B).

Considering the YSL actin ring is an essential force effector for epiboly progression^17^ and that its dynamics and shape are well known, we stained fixed embryos with phalloidin to highlight the actomyosin ring and zoomed in this particular region. By following YSL actomyosin ring evolution over time, we observed that transgenic embryos with higher number of asters displayed a wider YSL actin ring (typical of earlier epiboly stages) compared to wt siblings (Supplementary Fig. S2E–G). In conclusion, the YSL actomyosin ring in the transgenic embryos narrowed at a slow pace compared to wt embryos. We speculate that the delay in YSL actomyosin ring shrinking could cause the delay in epiboly.

Epiboly extension and the following stages up to 24 h of development were also tracked in different mRNA-injected embryos compared with wt embryos injected with DMSO. Sibling embryos were randomly divided into seven groups*.* We observed that 29% of *dclk2-gfp* RNA injected embryos already showed a delay on epiboly progression at the 50 pg dose, which become stronger with an increased dose (38% at 100 pg dose and 94% at 200 pg dose) (Fig. 2F and Supplementary Fig. S2C). Moreover, 100 pg injected embryos developed a shorter axis at 24 hpf, and the 200 pg dose injection produced body malformations and a complete lack of body axis extension in the most severe cases. Although 50 pg *DCX-gfp* RNA injected embryos are not visibly affected, above this dose they suffered a strong delay in epiboly and developmental defects apparent at 24 hpf (Supplementary Fig. S2D). Supplementary Figure S4 summarizes and compares the effect of Dclk2-GFP and DCX-GFP overexpressions and Dclk2-GFP transgenic embryos on YCL MT organization, its potential mechanistic cause and its consequences on epiboly progression and early development.

These experiments allow to conclude that, as long as the protein levels are kept in an appropriate range (stable transgenic line or low dose injection of the corresponding construct), the phenotype is compatible with development. Though induced, the phenotype displayed by the stable transgenic line can be seen as an example of plasticity and robustness in development.

### Dclk2-GFP YCL asters are transient 3D patterns of radially oriented MTs

**Figure 3:**
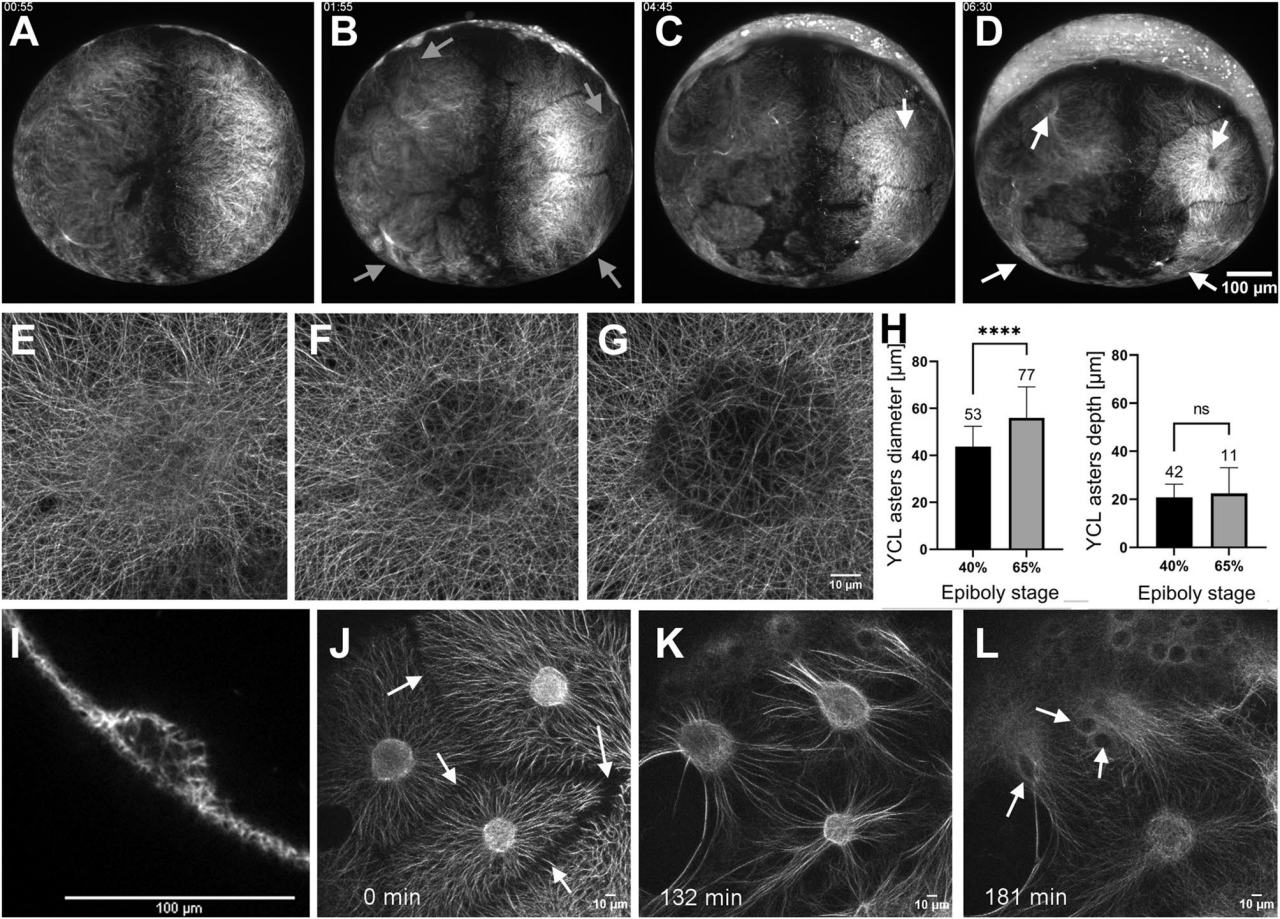
The YCL asters form and evolve throughout epiboly. (**A**–**D**) Embryo vegetal view. (**A**) Before epiboly, the MT network uniformly covers the exposed yolk. (**B**) At sphere stage, the MT network acquires a new configuration and rearranges into clear domains, with a central high dense MT bundle emanating from each of them (grey arrows). (**C**) Emerging from those bundles, defined MTs domains start migrating vegetally following a flat to hemisphere transition, leading to the formation of a visible YCL aster (white arrow) with radially oriented MT fibers in the middle of each domain (**D**) See Video S3. (**E**–**G**) High-resolution visualization of an aster formation through CSLM. See also Video S5. (**H**) After inspection of more than a hundred asters in different embryos we observe that, once formed, asters’ diameter increases with epiboly progression, while their depth remains constant. p-values: ****p < 0.0001, ns means p > 0.05. (**I**) A cross section of a YCL aster highlights its half-sphere shape. Aster’s dynamics are shown in Video S4 (**J**) The aster domains remain individualized with defined boundaries (white arrows), with clear opposing MT tips. (**K**) When the marginal blastoderm approaches the YCL asters (from the top left of the image), the MT network rearranges once again: thicker bundles are formed and reorient in the AV direction, and (**L**) the cores of the YCL asters actively migrate animalwards, concomitantly with eYSN undergoing epiboly (white arrows), until (**L**) they disappear underneath the YSL. See Videos S6 and S7.

To gain insight into the dynamics and the architecture of YCL asters, we performed LSFM and CLSM time-lapse analysis at different temporal and spatial resolutions (Fig. 3 and Supplementary Videos S3–S7). We could split the temporal YCL asters evolution into four steps: compartmentalization of yolk MTs into distinguishable domains; formation of asters; asters change of shape; and, finally, asters reabsorption below the approaching blastoderm.

When YSN become post-mitotic and epiboly starts, we observe a transition between a uniform yolk covering of MTs (Fig. 3A) to the formation of domains (Fig. 3B) apparently independent from each other (see also Videos S3 and S4). Subsequently, emerging from the blastoderm, YCL asters form as they migrate vegetally along the MT bundles anchored at the YSL (Fig. 3C). At 65% epiboly, just after the formation of the embryonic shield^9,44^, the e-YSN re-emerge from below the blastoderm and start moving towards the vegetal pole. At this moment, all the previously formed vegetal MTs compartments generate a clearly distinguishable round central region with MT bundles that are radially oriented from it (Fig. 3D). YCL asters span the yolk surface. In some cases, the e-YSN mitotic asters coexist with these newly formed YCL asters (Video S3).

In Fig. 3E–G and Video S5 we show, using high-resolution CLSM, the initial steps of one YCL aster formation. Before an actual YCL aster is formed, a dense central circle of disorganized MTs can be identified (Fig. 3E). Later on, the density of MTs in this central region is slowly reduced, evidenced by showing a weaker signal (Fig. 3F). Progressively a membrane depression forms the final YCL aster (Fig. 3G). At this point, the surrounding dense network of MTs orients radially from the center of the aster, and MT domains can be clearly distinguished (see Fig. 3J, white arrows).

A 3D analysis of the aster domain shows that asters change over time from a flat surface to a half-sphere shape (Supplementary Fig. S5 and Video S4) that is fully filled with thicker MTs bundles (see Fig. 3I). Moreover, analyzing more than 80 YCL asters, we found that the diameter of their representing hemispheres ranges from 43.72 ± 8.63 µm at 40% epiboly, to 55.93 ± 13.22 µm at 65% epiboly, therefore increasing significantly (p < 0.0001) with epiboly progression. Their depth instead remains constant (p = 0.987) ranging from 20.79 ± 5.52 at 40% epiboly to 22.48 ± 10.67 at 65% epiboly (Fig. 3H).

Zooming into the interaction area between asters, we observe clear boundaries between YCL asters domains (Fig. 3J) where MTs seem to repel each other. Once formed, the YCL asters persist on a quasi-static state and in the same position at the mesoscopic level of observation. Only when the blastoderm undergoing epiboly approaches, YCL asters dynamically move beneath the blastoderm, changing their shape (Fig. 3K,L and Video S6). We can observe a general displacement of the YCL aster towards the animal pole and a reorganization of the MTs in the aster, from a more uniform radial distribution to a biased preferred distribution (parallel to the AV direction of epiboly movement) (Fig. 3K,L and Supplementary Fig. S6). Soon after, the asters interact with the migrating e-YSN at the same time they are reabsorbed beneath the blastoderm (Fig. 3L and Videos S6 and S7).

### Dclk2-GFP YCL asters distribute in concentric rings in the yolk sphere

We have previously mentioned that while in the wt embryos long MT bundles run in the animal-vegetal direction and emanate from YSN-associated centrosomes, in the dclk2-GFP transgenic embryos the yolk MT network also exhibits multiple MT asters. In embryos with many asters we have noticed that the asters organize uniformly forming rings on the YCL at specific latitudes. In those, we observed the synchronous shape change and reabsorption of the asters belonging to the same ring. Rings of asters laying in more vegetal positions are sequentially reabsorbed, triggered by the proximity of the YSL and blastoderm undergoing epiboly. With the aim of determining if YCL asters really followed a particular pattern (symmetrical or biased in respect to some axis, for example) or instead if they located randomly, we calculated the spherical coordinates of the YCL asters, modeling the yolk as a sphere. Studying how its MT network is organized will give insights into how complex MT networks are influenced by a spherical geometry. LSFM offers the possibility to observe the embryo as a whole, as opposed to other microscopy techniques that produce partial information due to reduced spatial windows of observation.

**Figure 4:**
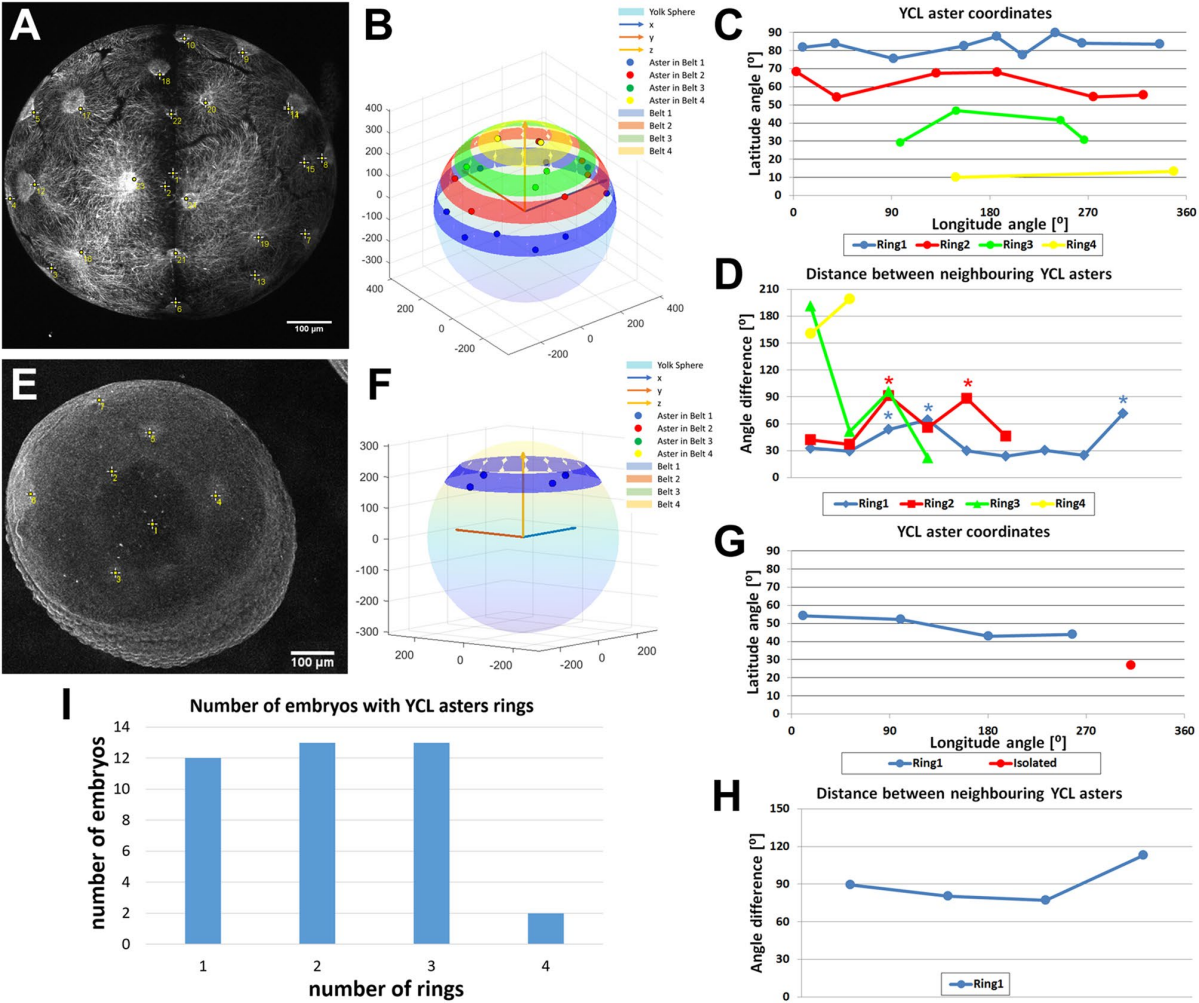
Analysis of the spatial YCL asters distribution in rings. (**A**) Maximum projection of a dclk2-GFP embryo where 22 YCL asters can be identified (yellow crosses) distributed in four rings. Identifiers 1 and 2 correspond to the vegetal pole and to the center of the embryo, respectively. (**B**) Projection of the aster position over the sphere modeling the yolk. Asters belonging to the same ring are represented by dots of the same color, rings are highlighted as belts at the calculated latitude. (**C**) YCL asters latitude (polar angle). YCL asters are not randomly located, but they group at four different latitudes (rings) (blue, red, green, and yellow). (**D**) Azimuthal angle differences between neighboring asters in rings 1 (blue), 2 (red), 3 (green), and 4 (yellow). Asters in this embryo are equidistant in 6/9 cases in ring1 and 4/6 cases in ring2. Cases with higher distances are marked with asterisks. (**E**–**H**) The same analysis for an embryo with only one YCL asters ring. Here 5 YCL asters can be identified: 4 asters distributed around 360°, and one in the vegetal pole. Each aster is highlighted with a yellow cross in Fig. 3E. Identifiers 1 and 2 correspond to the vegetal pole and to the embryo center, respectively. For this particular case, asters are equidistant (**H**). (**I**) Plot of the distribution of YCL asters in 1, 2, 3, or 4 rings. The analysis was performed on 40 embryos. (**B**,**F**) Created with MATLAB R2020b (https://www.mathworks.com).

The results are displayed in Fig. 4, where we show two representative embryos with four rings (Fig. 4A–D) and a single ring (Fig. 4E–H), respectively. The computation of their polar angles (latitude coordinates, ϑ) confirmed that asters indeed group at one or more specific latitudes (Fig. 4B,C,F,G), forming concentric rings around the animal-vegetal (AV) embryonic axis. We also calculated the angular distance between neighboring asters (Fig. 4D,H), showing that asters within a ring maintain a certain level of equidistance, mainly in the rings closer to the equator. To understand how common the different configurations within the population of transgenic embryos are, we quantified the number of rings in a sample of 40 embryos (Fig. 4I). The number of rings per embryo was highly variable, ranging from 1 to 4 rings. The number of asters within a ring was also variable and depended on the ring latitude, with rings close to the equator displaying more asters than rings closer to the vegetal pole.

As previously mentioned, YCL asters also form in conditions other than the transgenic line, where a stabilization of the MT network has been induced (i.e., dclk2-GFP overexpressing embryos, DCX-GFP overexpressing embryos, and in Taxol-incubated embryos). However, in these cases, YCL asters do not distribute in concentric rings but instead locate randomly in the yolk sphere. Only in the transgenic line YCL asters are almost perfectly distributed uniformly in the yolk surface and their location is radially symmetrical in the same direction that epiboly progression (see Fig. 4A and Supplementary Fig. S4). We speculate that the particular location of asters in rings in the transgenic line come from a level of regulation to which the transient mRNA is not subjected, and thus, asters form ectopically in random sites in the overexpressing embryos.

### MT polymerization and nucleation occur at YSN centrosomes and YCL asters

The above observations suggest that YCL asters could be acting as MTOCs. MTOCs are specific sites where MTs are nucleated and from where filaments grow through polymerization of α- and β-tubulin dimers^.^ Probably, the most well-known MTOC is the centrosome, used by cells during mitosis. However, in many non-dividing cells, the organization of the MTs is imparted by non-centrosomal MTOCs (nc-MTOCs), whose composition is rather unknown^45–48^. Nc-MTOCs are thought to contain a shorter list of proteins compared to centrosomal MTOCs, amongst which there have to be MT minus-end and plus-end interacting proteins recruited for growing MTs tips^49,50^. Therefore, to give YCL asters a molecular identity as MTOCs, we analysed the expression of two different proteins: EB3 and γ-tubulin, which complements the results on β-tubulin expression previously shown.

**Figure 5:**
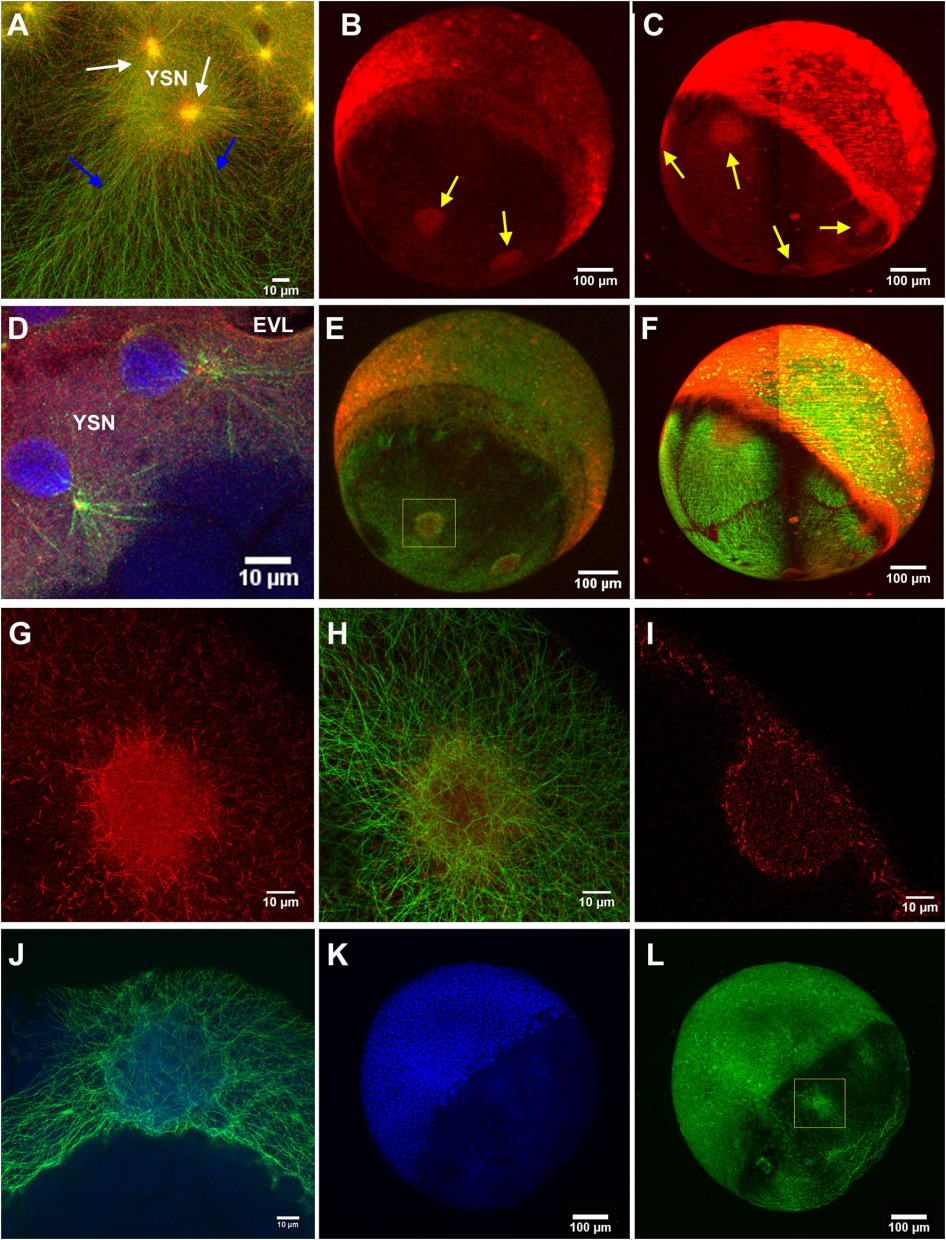
MT polymerization occurs at YSL and across the YCL*.* EB3-mCherry injected dclk2-GFP embryos allow us to simultaneously visualize MTs and MTs plus-ends. EB3-mCherry signal was found: (**A**) around e-YSN centrosomes (white arrows), as vegetalward oriented tracks in the YSL (blue arrows), and as scattered puncta all along the entire yolk cell [Merged signals, dclk2-GFP (green) and EB3-mCherry (red)]. See also Video S8. Whole imaging of the embryos using (**B**) CLSM and (**C**) LSFM also reveals the localization of EB3-mCherry signal at YCL asters (yellow arrows). (**D**) CLSM zoom in the YSL area to highlight YSL nuclei during division, in a EB3-mCherry injected dclk2-GFP transgenic embryo, fixed and stained with DAPI (blue). (**E**,**F**) Merged signals after adding the dclk2-GFP channel (green) to (**B**) and (**C**), respectively (red). (**G**,**H**) High resolution CLSM imaging of a YCL aster showing (**G**) EB3-mCherry signal and (**H**) its merge with the dclk2-GFP signal. (**I**) CLSM cross section of a YCL aster shows, through the EB3-mCherry signal, the MT polymerizing activity preferentially located at the periphery of the aster. (**J**–**L**) High resolution CLSM imaging of a YCL aster in a dclk2-GFP embryo, fixed and stained for DAPI (blue) at 60% epiboly. (**J**) corresponds to yellow inset in (**L**). No chromatin enriched structures are detected in YCL asters.

*EB3-mCherry* mRNA was injected at one-cell stage dclk2-GFP embryos to label MT plus-ends^51^. Its recruitment into growing MT tips was used as an indicator of the polymerization process during plus-end growth occurring at the yolk MTOCs. Thus, e-YSN centrosomes were marked by EB3 comets as newly polymerized plus ends emanate from them^52^ and extended further down along growing MTs (Fig. 5A and Video S8). EB3 localized to e-YSN centrosomes and the mitotic spindle throughout mitosis, as shown in Video S8. To counterstain nuclei, DAPI staining was performed on fixed 512–1000-cell stage dclk2-GFP embryos, previously injected with EB3-mCherry. Focusing in the YSL area, this experiment allows us to see the e-YSN nuclei, EB3-mCherry comets and dclk2-GFP highlighting the mitotic spindles of e-YSN divisions (Fig. 5A,D).

Interestingly, EB3 comets also spread towards the vegetal pole throughout the yolk cell and far from YSN centrosomes (Fig. 5B,C,E,F), which could indicate the non-centrosomal origin of some yolk MTs. In the case of the YCL asters, a diffuse EB3-mCherry signal was found at the core of every aster (Fig. 5B,C,E,F–I). Zooming into those asters (Fig. 5G,H) provides a picture of the distribution of EB3 puncta along MTs. EB3 comets are preferentially located in the aster’s periphery. This is consistent with a model proposed in^53^ where large asters have a spatial variation of polymerization rates, that are higher at the periphery. The cross-section of each of these centers reveals that EB3 distributes mainly in two opposed thin layers separated by a space with a lower density of EB3 signal (Fig. 5I), similar to the MT arrangement shown in Fig. 3I. In a parallel experiment, 60% Dclk2-GFP epiboly embryos were fixed and equally stained with DAPI. Dclk2-GFP is located in the asters (Fig. 5J,L), that instead do not exhibit DAPI signal (Fig. 5K). This is consistent with a non-centrosomic nature.

**Figure 6:**
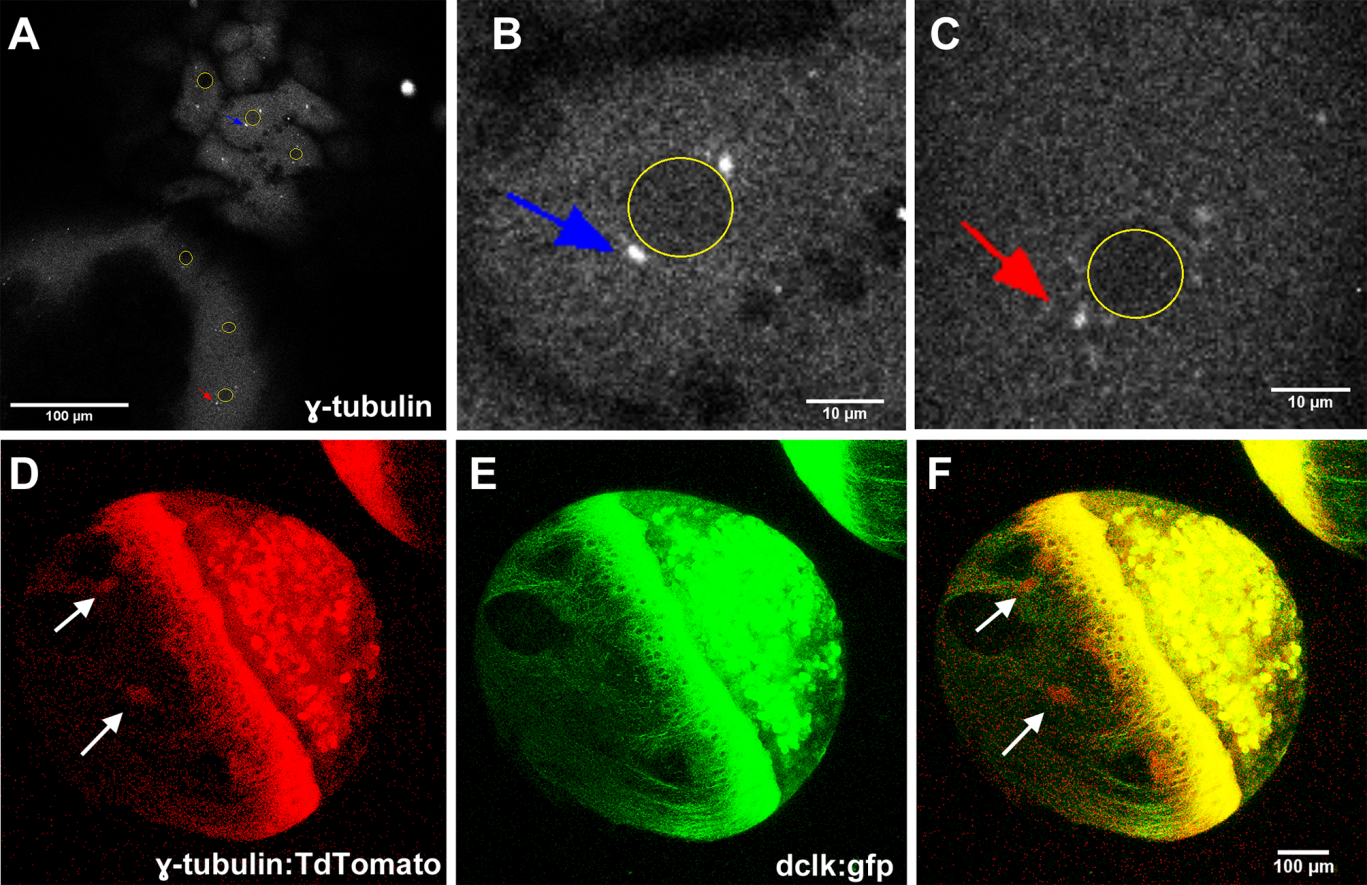
MT nucleation occurs at YSN centrosomes and YCL asters. γ-Tubulin expression can be observed through (**A**–**C**) immunostaining on fixed embryos and (**D**–**F**) in embryos coinjected with *γ-tubulin-TdTomato* mRNA and *dclk2-gfp* mRNA. γ-Tubulin signal has been found in (**B**) blastoderm centrosomes (blue arrow), (**C**) e-YSN centrosomes (red arrow) and (**D**–**F**) YCL asters colocalizing with dclk2-GFP signal (white arrows). In (**A**–**C**) the nuclei of both blastoderm and eYSN have been highlighted (yellow circles).

Nc-MTOCs have no centrosomes, but still MT nucleating capacity. In order to evaluate if YCL asters could have a nucleating capability, we decided to investigate γ-tubulin location. γ-Tubulin is a MT minus-end protein identified as one of the key nucleator components of MTOCs^49^. Although also found in nc-MTOCs, its function there as MTs nucleator, capper or stabilizer is not yet clear^48,50^. An immunostaining against γ-tubulin protein (Fig. 6A–C) allows confirming that both blastoderm (Fig. 6B) and e-YSN centrosomes (Fig. 6C) contain γ-tubulin. At later stages, when YCL asters are formed, the injection of *γ-tubulin-TdTomato* mRNA at one-cell stage dclk2-GFP embryos (Fig. 6D–F) reveals a high level of γ-tubulin protein at the core of every YCL aster, suggesting that yolk asters have MT nucleating ability.

In conclusion, the localization of EB3, γ-tubulin, and β-tubulin indicates that not only e-YSN centrosomes (found in the wt embryos and also in the dclk2-GFP transgenic embryos), but also dclk2-GFP YCL asters are sites of MT nucleation and growth.

## Discussion 

Here, we report evidence for the existence of a biological model of a vast MT network organized by means of multiple MT asters. This model is the yolk cell of the zebrafish dclk2-GFP transgenic line during epiboly stages. In this work, through the combination of LSFM and CSLM we deliver a detailed description of this MT network, which arises as a biological example for the organization of large MT networks in a spherical geometry.

In time, yolk MT asters (YCL asters) appear after epiboly’s initiation and are generated from an originally uniform MT network. YCL asters span across the yolk cell in variable numbers. These numbers are close to constant in a given female offspring, and after consecutive lays over time (Fig. 1I). We have followed up successive transgenic generations, confirming that the progenies are fertile and that the patterns are inherited. Interestingly, the MT organization by means of multiple YCL asters is not used by wt embryos but we managed to induce their formation in the wt condition by different approaches: dclk2-GFP/DCX-GFP overexpression and Taxol incubation (Fig. 2). These experiments have helped us understanding the potential mechanism underlying YCL aster formation in the transgenic line. We believe that an excess of dclk2 or DCX protein leads to an increase of MT stabilization and/or the formation of numerous MT nucleation sites in the yolk cell. Another set of experiments involving nocodazole injection in the yolk of the transgenic line supports the notion that YCL asters are formed by at least a population of stable MTs that are unsensitive to nocodazole effect (Supplementary Fig. S2). Regarding the mesoscopic arrangement, our data indicate that YCL asters in the transgenic line group at one or more latitudes, forming rings around the AV embryonic axis. This is a radially symmetrical layout without no apparent bias. Within a ring, they also keep a quasi-constant distance between them (Fig. 4). Interestingly, we have found a synchronicity in the dynamics of asters belonging to the same ring, evolving likewise through the different steps from aster formation to re-absorption when blastoderm undergoing epiboly approaches. Zooming into the asters, we have demonstrated that they are 3D structures, resembling hemispheres, that contain a radial disposition of MT bundles that changes as epiboly progresses (Fig. 3 and Supplementary Fig. S6) and that contain EB3 and γ-tubulin proteins, with EB3 preferentially located at the aster’s periphery (Figs. 5, 6). Regarding epiboly progression, embryos in the transgenic line with many asters show a delay with respect to control wt embryos and display a wider actin ring.

The above-mentioned results show how the yolk microtubule network of dclk2-GFP embryos arises as an in vivo model for the study of the collective behaviour of microtubules in a sphere. Despite the work done with the reconstitution of multiple large asters in a cell-free system^26,27,47^, to our knowledge there is no report of live non-artificial models forming big multiple MTOCs in an individual cell, with the exception of the transient formation of several small MTOCs in the immature mouse oocyte^39,54^. Surprisingly, we did not find YCL asters in wt eggs (non-injected, non-transgenic, non-Taxol induced), which suggests that our model of study is the result of an overexpression phenotype^55^ compatible with normal development as long as the gene is expressed at an appropriate range level. We believe that two main conclusions arise from this point. On the one hand, the variability observed is obviously remarkable from a developmental biology perspective (microtubule arrays can self-organize and adapt to external cues), and points to the yolk domain as a plastic and robust territory, able to present different configurations. On the other hand, our results prompt us to draw attention to the use of transgenic lines, since they do not always fully recapitulate the wt phenotype. In particular, our report might be useful to the scientific community, considering that this reporter line is one of the few available as a zebrafish in vivo microtubule reporter, and thus it is broadly used.

Our overexpression and pharmacological manipulation experiments are useful to understand the difference between the wt scenario and the transgenic line. This, together with the comparison between two of the conditions in which asters are formed (transgenic and mRNA overexpressing embryos, Supplementary Fig. S4), allow to gain an understanding of the MT dynamics and of the mechanisms underlying YCL aster formation. Our hypothesis is that YCL asters are present in the transgenic line due to an excess of Dclk2 protein, with a consequent overall increase of MT stabilization and/or nucleation in the yolk, and a correlation in epiboly delay when YCL asters appear in high number (Supplementary Fig. S2). Nocodazole results go toward the same direction and agree with works about astral MT populations in different scenarios^37,40,43^. Those MT populations are insensitive, or little affected, by nocodazole treatment because they are composed of at least a fraction of stable MTs.

From our point of view, though dispensable in the wt condition, YCL asters are functionally relevant in the transgenic line and might correspond to a gain-of-function phenotype as a consequence of gene dosage increase or genes being placed in a new context. Despite definitive functional experiments are missing, the above-mentioned assays set the basis to study YCL asters role in the near future. Also, our molecular identifiers and the lack of associated centrosomes allow us to classify them as non-centrosomal MTOCs. Thus, during these developmental stages, both centrosomal (e-YSN centrosomes) and non-centrosomal MTOCs (YCL asters) coexist in the yolk cell. Across the literature, it has been proposed that asters can form in diverse ways, both in natural or artificial conditions^56^. In vitro reconstitution experiments show the formation of individual asters in polymer-stabilized microfluidic droplets, by the molecular motor-mediated contraction of a spherical network^57^. In vivo, radial rearrangement of MTs in fish melanophore fragments is achieved through dynein-dependent MT nucleation^58^. The aster-type pattern allows to explore the intracellular space and to define the position of the organelles through interaction with MT motor proteins. We hypothesize that YCL asters are used for mechanical and structural support of the huge zebrafish yolk cell during epiboly, and for organelles and vesicles trafficking. The profound plasma membrane deformations identified with bright field microscopy (Fig. 1E and Video S4) indicate potential MTs anchoring sites, providing enough mechanical force to induce those deformations. In these sites, YCL asters would be serving for mechanical support. Indeed, it is known that cytoskeletal elements can pull membranes by polymerization or with the help of motor proteins^59,60^.

Despite being “artifactual”, this is an invaluable model for the organization of large MT networks in a spherical geometry, where MT asters sit on specific locations, around the AV axis. The synchronic dynamics of asters within the same ring, together with the radial symmetry around the AV axis prompted us to speculate about a global coordination mechanism and a geometrical and/or mechanical influence that direct asters position. The influence of shape in the distribution of asters will be tested in the future by manipulating embryos shape^17^. Currently, the yolk MT network of the Dclk2-GFP transgenic line is revealed as a biological model for the uniform distribution of many points in a (hemi)sphere, and could be a solution in nature for the classical best packing problem in spheres and minimal energy point configurations^61,62^. It would be very attractive to fit our biological data to computational 3D models^3^ that are able to simulate a variety of global orientations of the network upon changes in cellular shape or impact of external cues. We believe these models could help explain many of the experimental conditions analysed here (overexpression experiments, drug induction or transgenic female selection).

Finally, our results underscore the importance of using the appropriate imaging approach to observe the embryo as a whole. In the current work, LSFM supplies the access in vivo to the totality of the yolk MT network, it allows connecting events in time and space, and it reveals the phenotypic variability present in the population.

## Methods

Authors declare that all methods were carried out in accordance with relevant guidelines and regulations and in compliance with ARRIVE guidelines.

### Zebrafish strains

AB strain and Tg:(XlEef1a1:dclk2a-GFP) strain (from Marina Mione, CIBIO, University of Trento, Italy)^14^ were used. Animals were housed under standard conditions.

Zebrafish embryos were kept in E3 medium^63^ and staged as previously described^5^. Embryonic manipulations were done in E3 medium. The embryos analyzed in our study are always the result of outcrosses between Tg:(XlEef1a1:dclk2a-GFP) females with AB wt males. Up to 15 Tg:(XlEef1a1:dclk2a-GFP) females of successive generations were used in this work.

### DNA constructs and mRNA injections

The following expression constructs were used: EB3-mCherry^51^ and γ-tubulin-tdTomato, (kindly provided by Virginie Lecaudey, Goethe-Universität, Frankfurt); dclk2-GFP (kindly provided by Marina Mione, CIBIO, University of Trento) and DCX-GFP (kindly provided by Esteban Hoijman, CRG, Barcelona). mRNAs were synthesized using SP6 mMessage machine kit (Ambion, Life Technologies, Germany), after NotI linearization. Zebrafish embryos were injected using glass capillary needles (Harvard Apparatus 30-0020 GC100F-15) which were pulled with a needle puller (Sutter P-97) and attached to a microinjector system (World Precise Instrument PB820). Unless otherwise indicated, 100 pg of *EB3-mcherry* mRNA, *dclk2-gfp* mRNA, *DCX-gfp* mRNA or *γ-tubulin-tdTomato* mRNA were injected into 1-cell stage embryos.

### Pharmacological induction of YCL aster formation

Wt embryos at sphere stage were manually dechorionated and incubated in egg water containing 5, 25, 40, or 50 μM Taxol (Sigma, T7191) (DMSO diluted). In our hands, concentrations higher than 50 μM precipitated, and therefore were not included in this study. The embryos were exposed to the pharmacological treatment until desired stages and then were fixed for subsequent β-tubulin immunostaining. Control embryos were treated with equivalent amounts of DMSO, with no adverse effect on development.

### Functional assay of MT dynamics with nocodazole

1 nl (5 pg/nl) of nocodazole was injected in the YSL at 1000-cell stage in Dclk2-GFP embryos. Immediately after, embryos were mounted and observed with LSFM and CLSM. A control group was injected with an equivalent amount of DMSO.

### Whole-mount immunohistochemistry

Mouse anti-β-tubulin antibody (E7, Developmental Studies Hybridoma Bank, DSHB) was used at 1:200, and mouse anti-γ-tubulin antibody (T5326 Sigma-Aldrich) was used at 8 μg/ml. The secondary antibody was in-house conjugated^64^ to the Abberior STAR 635P fluorophore (Sigma) and used at 8 μg/ml.

γ-Tubulin staining was performed as previously described^21^. Briefly, embryos were fixed in 4% PFA overnight at 4 °C. Fixed embryos were washed several times in PBS and dechorionated. Permeabilization was done in 0.3% Triton X-100 (in PBS) for 1 h, exchanging buffer every 15 min. Blocking was performed in blocking solution (1% BSA in 0.3% Triton X-100 in PBS) for 2 h, at room temperature (RT). Embryos were incubated with γ-tubulin primary antibody in blocking solution overnight at 4 °C. After washing the embryos in 0.3% Triton X-100 in PBS over a day, they were incubated in the secondary antibody in blocking solution overnight at 4 °C. After 3–4 washes in PBS, the embryos were ready to be mounted and imaged.

β-Tubulin antibody staining was performed as previously described^65^ with some modifications. Briefly, embryos were dechorionated and fixed in MT assembly buffer (80 mM KPIPES (pH 6.5), 5 mM EGTA, 1 mM MgCl_2_, 3.7% formaldehyde, 0.25% glutaraldehyde, 0.5 µM Taxol, and 0.2% Triton X-100) for 6 h at RT. Fixed embryos were dehydrated and kept in methanol at − 20 °C overnight, or for several days. After, they were washed several times in PBS containing 0.1% NP40, for re-hydration. Re-hydrated embryos were then incubated in 100 mM NaBH4 in PBS for 6–16 h at RT and washed extensively in Tris buffered saline (TBS). Blocking was performed in blocking solution (2% BSA in TBS) for 30 min at RT. Embryos were incubated with β-tubulin primary antibody in blocking solution overnight at 4 °C. After washing them 4–5 times in TBS, they were incubated in the secondary antibody in blocking solution for 2–3 h at RT. After 3–4 washes in TBS, they were ready to be mounted and imaged.

### Nuclei and actin stainings

Zebrafish embryos were fixed overnight in 4% PFA/PBS at 4 °C. After several short PBS washes, they were dechorionated. Permeabilization and blocking was achieved with several short PBST washes (PBS and 0.3% tritonx100) and a 2-h Incubation step with 1% BSA in PBST. Embryos were incubated for 1 h with DAPI (1:500) (A1306, Invitrogen) for nuclear staining and phalloidin-647 (1:500) (A2287, Thermo Fisher) for actin staining. After few short PBS washes, they were ready to be mounted for CLSM Imaging.

### Imaging setups and sample preparation for live and fixed imaging

CLSM was performed on a commercial Leica SP8 equipped with a supercontinuum white-light laser, a 405 nm laser and Hybrid detectors and with HC PL APO CS2 10 ×/0.40 DRY and HC PL APO CS2 63 ×/1.40 OIL objectives. For live imaging, dechorionated embryos were mounted in 0.5% low melting point agarose (LMPA) (Promega, V2111) in E3 medium, on glass-bottom dishes (MatTek). Fixed samples were mounted in 1% LMP agarose in PBS, on glass-bottom dishes (MatTek) (Supplementary Fig. S1C).

LSFM imaging was performed using a custom-made light-sheet set up^15^, called Flexi-SPIM. For illumination, we used 488, 561 and 637 nm lasers (Cobolt, MDL488, MLD561, MLD637) and two air objectives (Nikon 4 ×, NA 0.13). For fluorescence detection, we used water dipping objectives (Nikon 10 ×, NA 0.3 and 20 ×, NA 0.5), filters, and a sCMOS camera (Hamamatsu OrcaFlash4 v2). Thanks to our configuration, multiple views of the specimen can be visualized, providing in-toto embryo representations. We realized, along the experiments, that LSFM imaging of the yolk leads to image degradation along the illumination axis. This is due to light refraction on the lipid-filled spherical yolk cell, which acts as a lens. Sequential side illumination, although requiring a simple fusion process, increases image quality. However, this leads to a blind central region on the data sets. This effect is neglectable in the blastoderm. Embryos were simply mounted, without removing their chorion, within either a 1.5% LMP agarose cylinder or a 1 mm inner diameter FEP tube filled with E3 medium (Supplementary Fig. S1A,B).

Bright-field images were acquired with a stereoscope (Nikon SMZ800) or the LSFM set up and a source of white-light, bulb or led, respectively.

### Image analysis

For the quantification of the YCL asters number (Fig. 1I), we manually counted the number of YCL asters on the Z maximum projection of LSFM and LSCM images. Only the eggs offering a vegetal view (the totality of the YCL asters could be accessed) and eggs offering a lateral view, in which radial symmetry could be assumed, were considered. The analysis includes 9 females in total.

To evaluate YCL asters sizes (Fig. 3H), the 3D z-stacks were analyzed. YCL MT asters’ depth and diameter were measured with Fiji^66^ in their max value.

To determine YCL MT aster distribution over the yolk (Fig. 4), 3D stacks acquired either by LSFM or CLSM were analysed. Images relative to a single time point were used, correspondent to the embryo at 50–65% epiboly, i.e., when the YCL asters are clearly visible. A Fiji custom-made macro permits to select and export the 3D coordinates of the centre of the embryo, the vegetal pole, and the asters. Elaborating these 3D coordinates through a Matlab script, the AV axis orientation and the radius of the modelled yolk sphere are obtained. Based on this, a rigid body transformation of all the points is applied so that the centre of the embryo is coincident with the coordinate origin, and the AV axis is aligned along the “z” axis. Describing the YCL MT aster coordinates by mean of spherical coordinates the latitude (polar angle relative to the AV axis) and the longitude (azimuthal angle) of each of the MT asters are computed. For each embryo, MT asters having a similar latitude (i.e., a maximum of 20° of difference) were considered to belong to the same ring, concentric to AV axis. For each ring, the differences between longitudes of neighbour MT asters were calculated to examine eventual angular equidistance between them. Equidistance is defined as the angular distances' differences is belonging to a 20° range. In cases where equidistance is not found, we suspect an aster in between could not be detected from the image 3D field of view.

The thickness of MT bundles (N = 17) in 40 μM taxol-treated embryos was obtained from confocal Z maximum projections of Dclk2-GFP transgenic embryos. Briefly, a straight line perpendicular to the bundle and positioned at half way of its length provided the average thickness of the bundle.

### Statistical analysis

Statistical analysis for Fig. 3H was performed using GraphPad Prism 9. Two-tailed p-values between YCL asters’ size (diameter and depth) at 40% and 65% epiboly were estimated through the Mann–Whitney test.

### Animal welfare

The protocol with CEEA ref. MMT-21-0033-P1 which is used in this project, has been approved by the Animal Research Ethics Committee (CEEA) from Barcelona Biomedical Research Park (PRBB) according to the European Union regulations regarding work protocols in experimental animal models.

## Supplementary Information

Supplementary Information.

Supplementary Video S1.

Supplementary Video S2.

Supplementary Video S3.

Supplementary Video S4.

Supplementary Video S5.

Supplementary Video S6.

Supplementary Video S7.

Supplementary Video S8.
