# Supplementary material for: Multiple asters organize the yolk microtubule network during dclk2-GFP zebrafish epiboly
Source: Sci Rep. 2022 Mar 8;12:4072. doi: 10.1038/s41598-022-07747-7 (PMC8904445; doi:10.1038/s41598-022-07747-7)
Supplement: Supplementary file 1 — Supplementary Information. [file 41598_2022_7747_MOESM1_ESM.pdf]

MULTIPLE ASTERS ORGANIZE THE YOLK MICROTUBULE NETWORK DURING DCLK2-GFP ZEBRAFISH EPIBOLY

Supplementary Material:

**Supplementary Figure S1**	Scheme of the different mounting protocols for LSFM and LSCM
**Supplementary Figure S2**	YCL aster formation and their impact on early development in transgenic and dclk2-gfp and DCX-gfp mRNAs overexpressing embryos.
**Supplementary Figure S3**	Effect of yolk-nocodazole injection on dclk2-GFP transgenic embryos
**Supplementary Figure S4**	Schematic comparison between the two genetic conditions that can produce YCL asters
**Supplementary Figure S5**	3D evolution of an aster over time
**Supplementary Figure S6**	Orientation of the MT bundles within representative YCL asters regarding distance to blastoderm margin
**Supplementary Note S1**	Image analysis tools

**Supplementary Video 1**	3D render of a dclk2-GFP transgenic half-embryo exhibiting multiple YCL asters
**Supplementary Video 2**	Progression during epiboly of a nocodazole-treated embryo.
**Supplementary Video 3**	*In-toto* MT dynamics of a dclk2-GFP embryo during epiboly
**Supplementary Video 4**	3D render of the YCL aster progression over time
**Supplementary Video 5**	First steps of YCL aster formation in a dclk2-GFP embryo
**Supplementary Video 6**	Change of shape and disappearance of YCL asters underneath the blastoderm undergoing epiboly in a dclk2-GFP transgenic embryo.
**Supplementary Video 7**	Disappearance of YCL asters in a dclk2-GFP embryo
**Supplementary Video 8**	Dynamics during e-YSN division in a dclk2-GFP embryo

Supplementary Figure S1: Scheme of the different mounting protocols for LSFM and LSCM

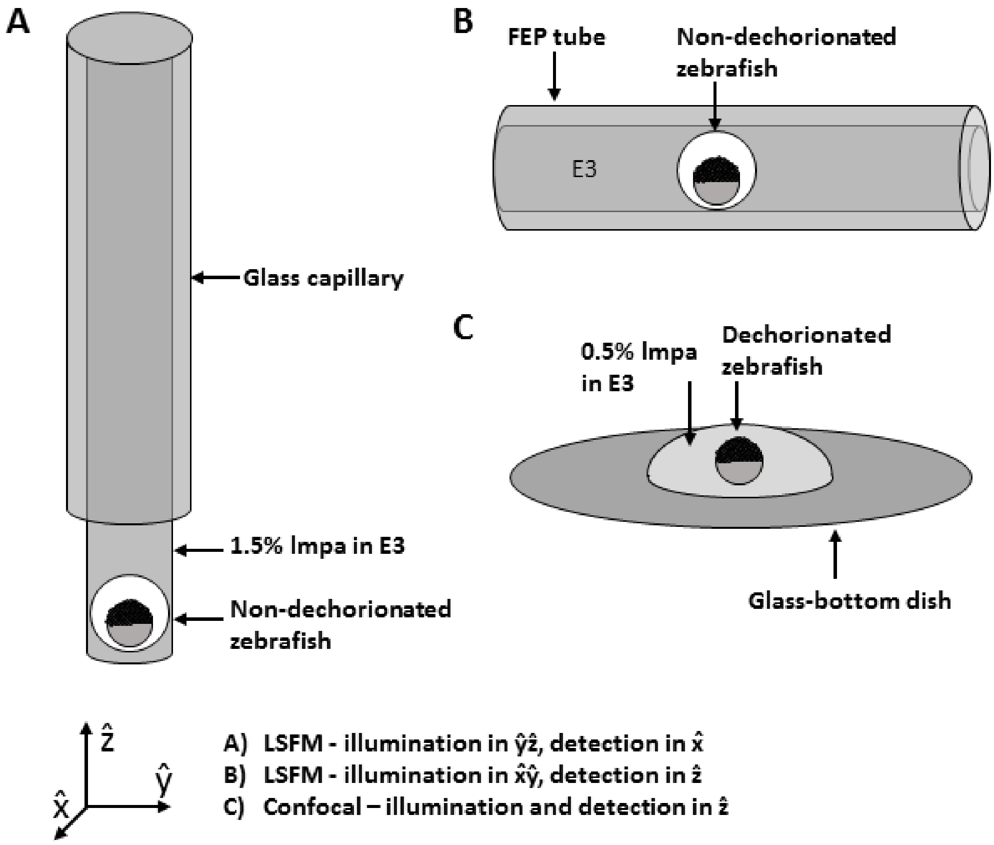

Supplementary Figure S1: Scheme of the different mounting protocols for LSFM and LSCM. Non-dechorionated embryos are either **(A)** embedded in a 1.5% lmpa cylinder extruded from a glass capillary, or **(B)** inserted in an E3-filled FEP tube, also used for transporting the sample toward the field of view of the microscope, in the LSFM approach. **(C)** In CLSM, dechorionated embryos are embedded in 0.5% lmpa, and imaged through a glass-bottom dish. In the bottom part, the coordinate system explains the illumination and detection directions of for each configuration.

Supplementary Figure S2: YCL aster formation and their impact on early development in transgenic and *dclk2-gfp* and *DCX-gfp* mRNAs overexpressing embryos.

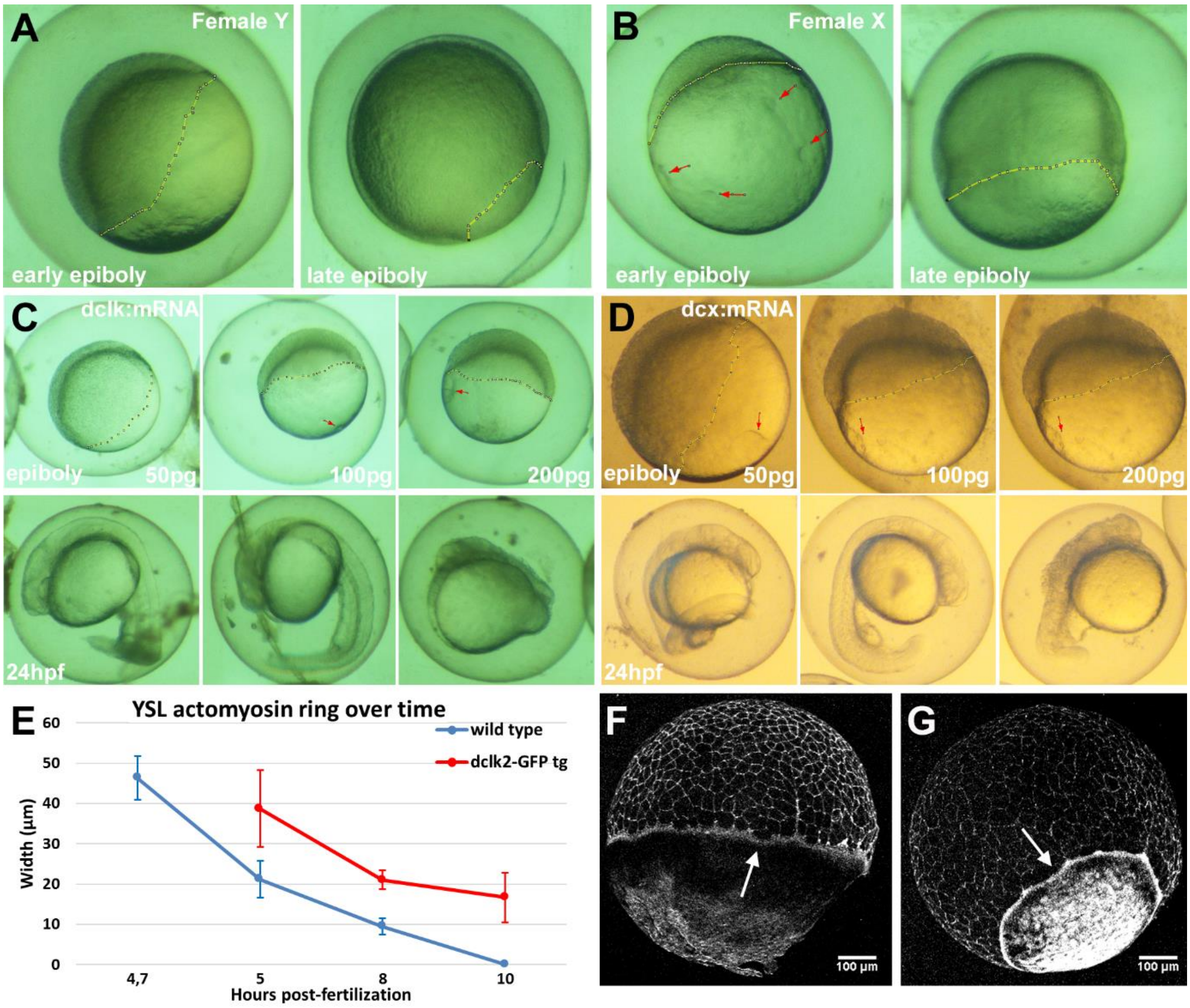

Supplementary Figure S2: YCL aster formation and their impact on early development in transgenic and *dclk2-gfp* and *DCX-gfp* mRNAs overexpressing embryos. **(A-B)** epiboly stage comparison from BF Imaging of representative sibling embryos from different Tg dclk2-GFP females. **(A)** female Y, low number of asters, (10 eggs analyzed) and **(B)** female X, medium number of asters, (10 eggs analyzed) at early (left) and late (right) epiboly stages (epiboly extension: dashed yellow line. YCL asters: red arrows) **(C-D)** epiboly stage comparison from BF imaging of representative sibling embryos, injected with increasing *dclk2-gfp* mRNA or *DCX-gfp* mRNA doses. **(C)** increasing doses of *dclk2-gfp* mRNA mRNA injections partially recapitulate the transgenic phenotype. Upper row: increasing mRNA doses proportionally induce a delay in epiboly (dashed red line) (Wt embryos, N=30; 50pg-injected embryos, N=20; 100pg-injected embryos, N=35; 200pg-injected embryos, N=20). Ectopic asters (red arrows) are formed from 100pg dose. Lower row: development follow up at 24hpf. **(D)** Equivalent experiment with *DCX-gfp* mRNA injections produces asters (upper row, red arrows) and developmental defects (lower row) starting at lower doses compared to experiment in (C). **(E)** Actomyosin ring contraction is delayed in dclk2-GFP transgenic embryos with many asters, compared to wt siblings. Embryos were fixed at 4.7hpf (wt embryos N=5), 5.3hpf (wt embryos N=7, Tg embryos N=3), 8hpf (wt embryos N=5, Tg embryos N=5) and 10hpf (wt embryos N=5, Tg embryos N=10) and stained with phalloidin. Standard Deviations are shown. **(F)** CLSM whole-embryo maximum projections of phalloidin stained dclk2-GFP transgenic embryos and **(G)** wt control siblings. At 8hpf, wt control embryos are at 75% epiboly and dclk2-GFP transgenic embryos are at 50% epiboly. Arrows indicate the formation of the actomyosin ring, ahead of the advancing blastoderm.

Supplementary Figure S3: Effect of yolk-nocodazole injection on dclk2-GFP transgenic embryos

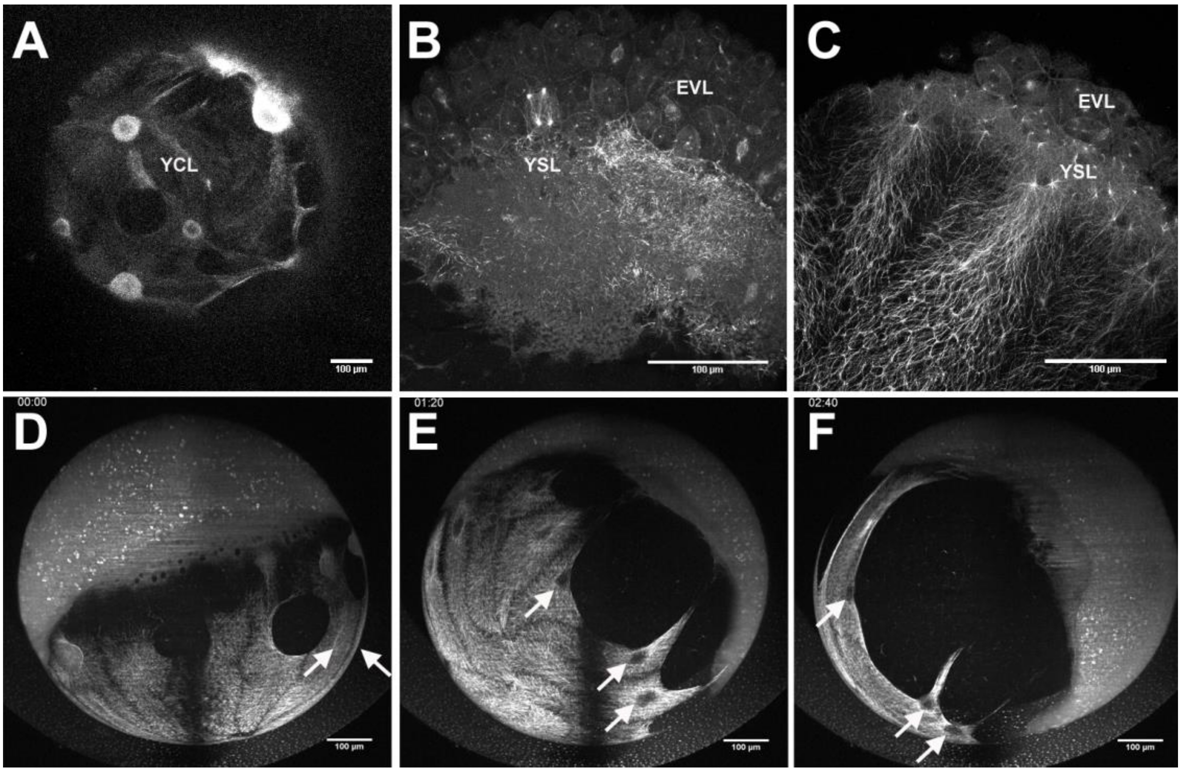

Supplementary Figure S3: Effect of yolk-nocodazole injection on dclk2-GFP transgenic embryos. **(A)** Vegetal view. YCL asters persist after nocodazole injection. (**B**) YSL region of a nocodazole-treated embryo, showing the disruption of both the MT mesh around the YSN and the AV MT arrays. **(C)** Instead, in control DMSO-injected embryos, MT arrays in the YSL are unaffected. **(A-C)** Acquired with high resolution CLSM. **(D-F)** LSFM time-lapse imaging of the progression during epiboly of a nocodazole-treated embryo. Notice YCL asters persistence (white arrows). See also Video S2. Scale bar: 100 µm.

Supplementary Figure S4: Schematic comparison between the two genetic conditions that can produce YCL asters

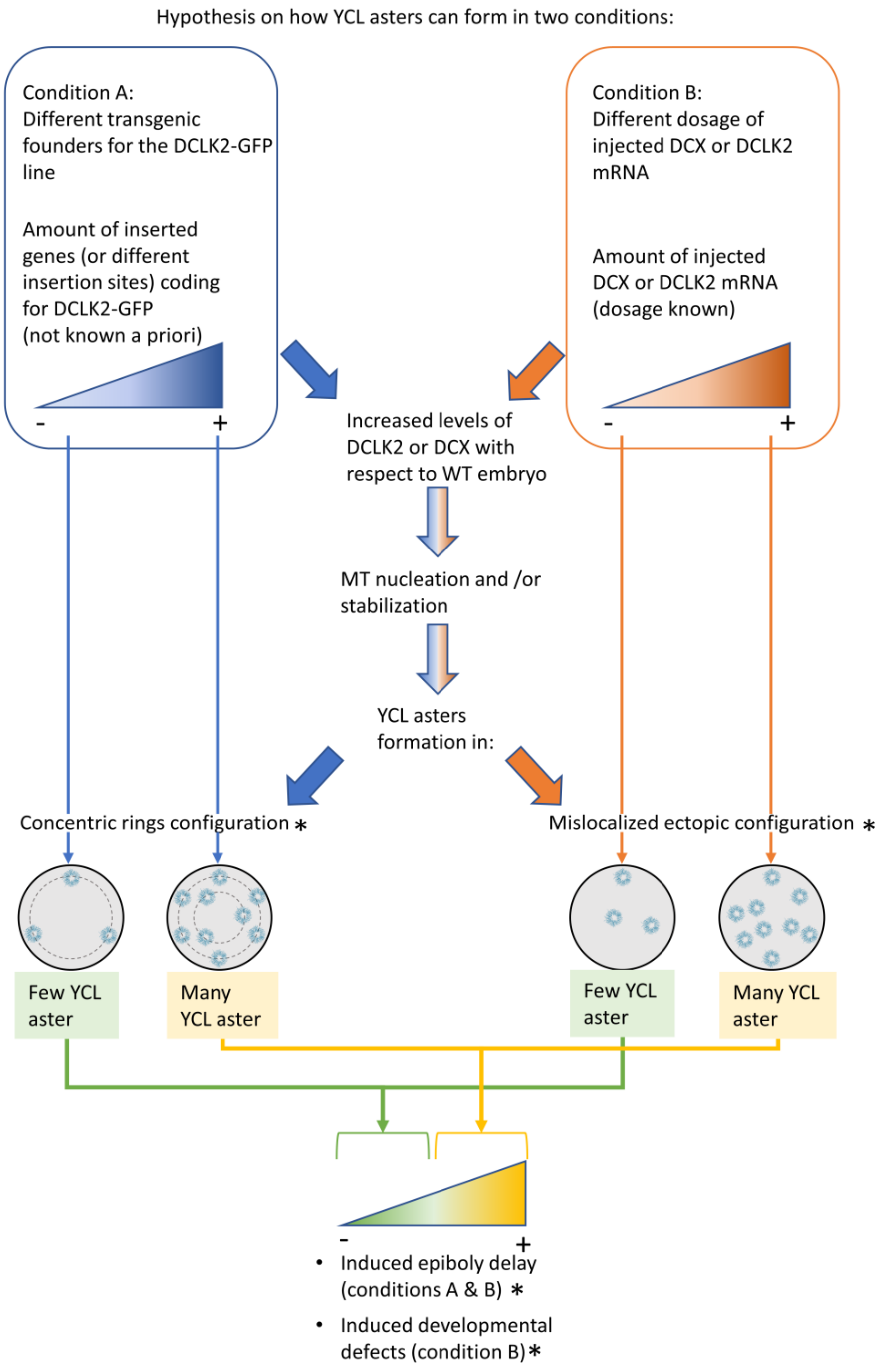

Supplementary Figure S4: Schematic comparison between the two genetic conditions that can produce YCL asters. Condition A: dclk2-GFP stable transgenic line and **Condition B:** transient expression of dclk2-GFP or DCX-GFP mRNA constructs. The graph summarizes YCL MT organization, its potential mechanistic cause, and its consequences on epiboly progression and early development in the two conditions. Asterisks highlight steps in the cascade for which we have no explanation yet: 1) what drives aster formation in concentric rings in the Tg line, as opposed to a random location in the overexpressing embryos, 2) why embryos with many asters have epiboly delay, and 3) why embryos with higher mRNA doses have epiboly delay and developmental defects.

Supplementary Figure S5: 3D evolution of an aster over time

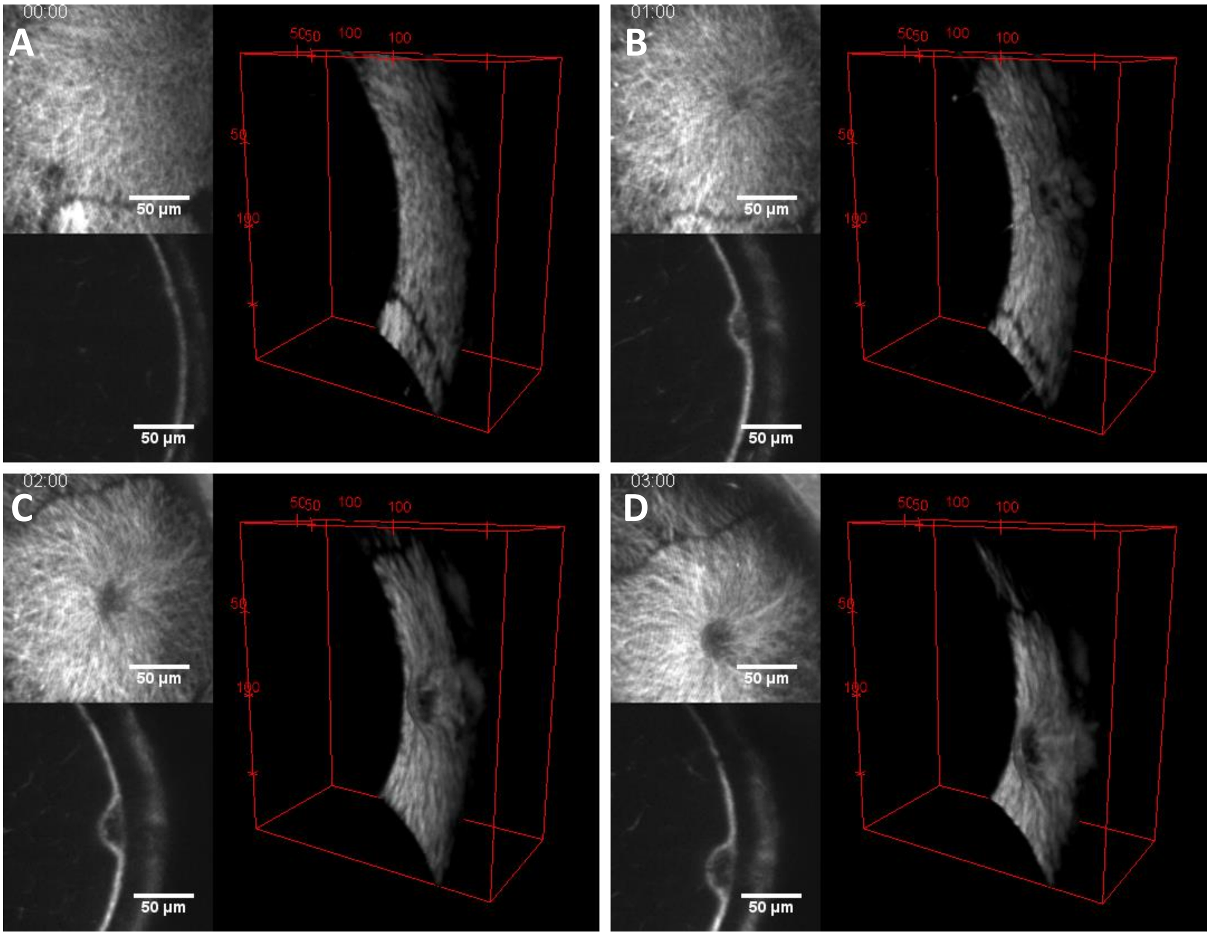

Supplementary Figure S5: 3D evolution of an aster over time. (A-D) Four time points (at 0, 1, 2 and 3 hours) of the evolution of one aster of the embryo displayed in Figure 4 A-D (and Supplementary Video S3). On each time point we show: the maximum projection of the region of interest (upper left corner); a cross section at the plane with maximum aster depth (lower left corner); and a 3D projection of the membrane depression created by the aster (right hand). The 3D analysis shows that asters change it shape over time from a flat surface to a half-sphere shape. Scale bar: 50 µm. Full movie available in Supplementary VideoS4.

Supplementary Figure S6: Orientation of the MT bundles within representative YCL asters regarding distance to blastoderm margin

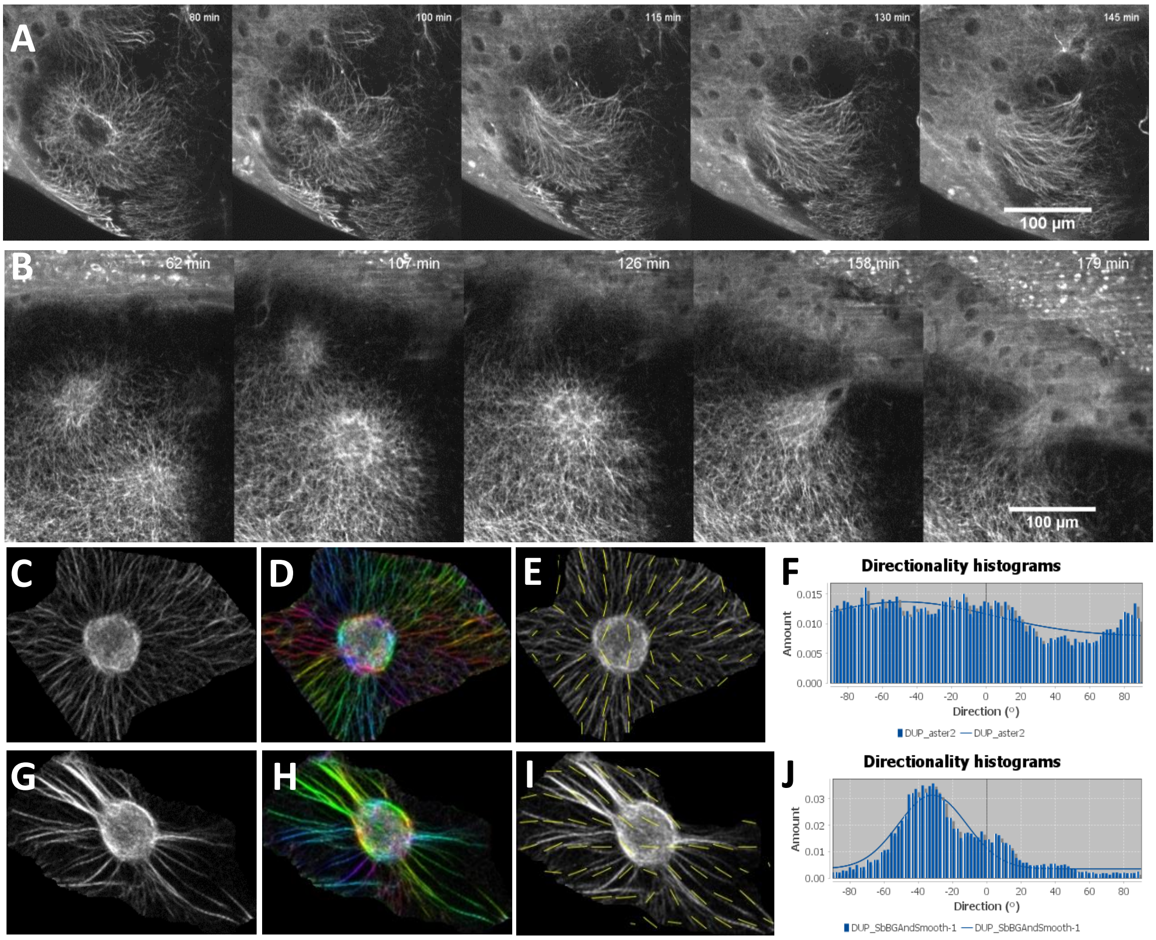

Supplementary Figure S6: Orientation of the MT bundles within representative YCL asters regarding distance to blastoderm margin. **(A)** and **(B)** show two examples of YCL aster reabsorption as the blastoderm margin approaches. To estimate the local orientation of the MT bundles we used two ImageJ plug-in: OrientationJ and Directionality. **(C-F)**: YCL asters not adjacent to blastoderm margin show an isotropic distribution of MT bundles. **(C)** Original image of YCL aster. **(D)** OrientationJ Analysis module performs a visual representation with a color map of the distribution of the MT bundles. **(E)** Vector field overlaid on the original image, performed by OrientationJ Vector Field module. **(F)** Histogram computed with Directionality plugin, indicating the amount of MT bundles in a given direction. This flat histogram indicates a very isotropic MT content in this type of YCL asters. **(G-J)** The same analysis was performed when the blastoderm margin approaches the YCL asters. **(J)** The histogram shows a distinguishable peak, indicating a preferred orientation of the MT bundles in the AV direction. Scale bar:100 µm.

Supplementary Note S1: Image analysis tools

To measure the width of actomyosin ring (Supplementary Figure S2), confocal surface projections at different embryo stages were obtained with Fiji[1] and mean width and standard deviations were plotted (Excel, MS Office).

To display the evolution of the 3D structure of the aster domain over time (Supplementary Figure S5), we used Fiji 3D Viewer[2].

To estimate the local orientation of the MT bundles in YCL asters (N=9) at different distances from the YSL (Supplementary Figure S6), we used two Fiji plug-ins: OrientationJ[3] and Directionality (created by Jean-Yves Tinevez, Institute Pasteur). We used the OrientationJ Analysis functionality to render a visual representation of the orientation of the MTs in the YCL asters, and the functionality OrientationJ Vector Field to create a vector field map of the selected images. To generate the histograms that show the amount of MT bundles in particular directions the local gradient orientation method of Directionality plugin was used. The background was subtracted and a smooth filter was applied (through FIJI) in the original images before the orientation analysis was performed.

References

1. Schindelin, J. et al. Fiji: an open-source platform for biological-image analysis. Nat. Methods 9, 676–682 (2012).
2. Schmid, B., Schindelin, J., Cardona, A., Longair, M., & Heisenberg, M. A high-level 3D visualization API for Java and ImageJ. BMC Bioinformatics, 11(1) (2010).
3. Püspöki, Z., Storath, M., Sage, D. & Unser, M. Transforms and operators for directional bioimage analysis: A survey. *Adv. Anat. Embryol. Cell Biol.* (2016).

Video Legends:

Supplementary VideoS1: 3D render of a dclk2-GFP transgenic half-embryo exhibiting multiple YCL asters. Related to Figure 1.

Supplementary VideoS2: Progression during epiboly of a nocodazole-treated embryo. LSFM time-lapse images were acquired with alternate dual side illumination and single side detection, allowing to visualize half embryo sphere. The position of the asters is highlighted by white arrows. The embryo has been imaged within its chorion. The total movie (5 hours) consists of 60 time points every 5 minutes. Scale bar 100 µm. Related to Supplementary Figure 3.

Supplementary VideoS3: *In-toto* MT dynamics of a dclk2-GFP embryo during epiboly. LSFM time-lapse images were acquired with simultaneous dual side illumination and double side detection, allowing to visualize the whole embryo sphere: vegetal view on the left, animal view on the right. YCL asters are highlighted with white arrows. The total movie (14 hours) consists of 168 time points every 5 minutes. Scale bar 100 µm. Related to Figure 4.

Supplementary VideoS4: 3D render of the YCL aster progression over time. For each time point we show: the maximum projection of the region of interest (upper left corner); a cross section at the plane with maximum aster depth (lower left corner); and a 3D projection of the membrane depression created by the aster (right hand). The total movie (5 hours) consists of 60 time points every 5 minutes. Scale bar 50 µm. Related to Figure 4 and Supplementary VideoS3.

Supplementary VideoS5: First steps of YCL aster formation in a dclk2-GFP embryo. Acquired using CLSM. The total movie (1.5 hours) consists of 616 time points every 8 seconds. Scale bar 10 µm. Related to Figure 4.

Supplementary VideoS6: Change of shape and disappearance of YCL asters underneath the blastoderm undergoing epiboly in a dclk2-GFP transgenic embryo. Acquired using CLSM. The total movie (4 hours) consists of 240 time points every minute. Scale bar 10 µm. Related to Figure 4.

Supplementary VideoS7: Disappearance of YCL asters in a dclk2-GFP embryo. LSFM time-lapse images were acquired with alternate dual side illumination and single side detection, allowing to visualize half embryo sphere. The total movie (~7 hours) consists of 83 time points every 5 minutes. Scale bar 100 µm. Related to Figure 4.

Supplementary VideoS8: Dynamics during e-YSN division in a dclk2-GFP embryo. Acquired using CLSM. The different panels show: EB3 (red, left panel); MT (green, center panel); and merged signals (right panel). The total movie (49 minutes) consists of 33 time points every 89 seconds. Scale bar 10 µm. Related to Figure 5.
